# Life expectancy and mortality in 363 cities of Latin America

**DOI:** 10.1038/s41591-020-01214-4

**Published:** 2021-01-25

**Authors:** Usama Bilal, Philipp Hessel, Carolina Perez-Ferrer, Yvonne L. Michael, Tania Alfaro, Janeth Tenorio-Mucha, Amelia A. L. Friche, Maria Fatima Pina, Alejandra Vives, Harrison Quick, Marcio Alazraqui, Daniel A. Rodriguez, J. Jaime Miranda, Ana V. Diez-Roux, the SALURBAL group, Marcio Alazraqui, Hugo Spinelli, Carlos Guevel, Vanessa Di Cecco, Adela Tisnés, Carlos Leveau, Adrián Santoro, Damián Herkovits, Andrés Trotta, Patricia Aguirre, Santiago Rodríguez López, Natalia Tumas, Nelson Gouveia, Maria Antonietta Mascolli, Anne Dorothée Slovic, Lucas Soriano Martins, Cláudio Makoto Kanai, Mauricio Barreto, Gervásio Santos, Anderson Dias de Freitas, Caio Porto De Castro, José Firmino de Sousa Filho, Maria Izabel dos Santos Bell, Roberto Fernandes Silva Andrade, Leticia Cardoso, Mariana Carvalho de Menezes, Maria de Fatima de Pina, Daniel Albert Skaba, Joanna Miguez Nery Guimarães, Vanderlei Pascoal de Matos, Waleska Teixeira Caiaffa, Amélia Augusta de Lima Friche, Amanda Cristina de Souza Andrade, Camila Teixeira Vaz, Débora Moraes Coelho, Denise Marques Sales, Guilherme Aparecido Santos Aguilar, Julia de Carvalho Nascimento, Lídia Maria de Oliveira Morais, Mariana de Melo Santos, Uriel Moreira Silva, Patricia Frenz, Tania Alfaro, Cynthia Córdova, Pablo Ruiz, Mauricio Fuentes, Marianela Castillo, Sebastian Pedrero, Lorena Rodríguez, Tamara Doberti, Alejandra Vives Vergara, Alejandro Salazar, Andrea Cortinez-O’Ryan, Cristián Schmitt, Francisca Gonzalez, Fernando Baeza, Flavia Angelini, Laura Orlando, Olga Lucía Sarmiento, Diana Higuera, Catalina González, Felipe Montes, Andres F. Useche, Oscar Guaje, Ana Maria Jaramillo, Luis Angel Guzmán, Diego Lucumí Cuesta, John Alexis Guerra, Jorge Alexander Bonilla, Luis Angel Guzman, Mario Linares, Philipp Hessel, Ricardo Morales, Camilo Triana, Maria Alejandra Wilches, Alejandro Palacio, Fabian Camilo Peña, Joaquín Hernando Jaramillo Sabogal, Julieth Lopez, Karen Fajardo, Marcelo Botero, Natalia Cely, Paola Martinez, Carlos Moncada, Jose David Meisel, Eliana Martinez, María Fernanda Kroker-Lobos, Manuel Ramirez-Zea, Monica Mazariegos, Analí Morales, Tonatiuh Barrientos-Gutierrez, Carolina Perez-Ferrer, Javier Prado-Galbarro, Nancy Paulina López-Olmedo, Filipa de Castro, Rosalba Rojas-Martínez, Alejandra Jauregui, Dalia Stern, Horacio Riojas, José Luis Texcalac, Desirée Vidaña Pérez, J. Jaime Miranda, Akram Hernández Vásquez, Francisco Diez-Canseco, Lorena Saavedra Garcia, Ross Hammond, Daniel Rodriguez, Iryna Dronova, Xize Wang, Mika Moran, Yuanyuan Zhao, Yang Ju, Xavier Delclòs-Alió, Peter Hovmand, Ellis Ballard, Jill Kuhlberg, Ana V. Diez-Roux, Amy Auchincloss, Sharrelle Barber, Usama Bilal, Felipe Garcia-España, Brent Langellier, Gina Lovasi, Leslie McClure, Yvonne Michael, Kari Moore, Ana Ortigoza, Harrison Quick, D. Alex Quistberg, Brisa N. Sanchez, Ivana Stankov, Jose Tapia-Granados, Goro Yamada, Jordan Rodriguez-Hernandez, Steve Melly, Ione Avila-Palencia, Josiah Kephart, Pricila Mullachery, Bricia Trejo, Ariela Braverman, Dustin Fry, Rosie Mae Henson, Kevin Martinez-Folgar, S. Claire Slesinski, Katherine Indvik, Andrea Bolinaga

**Affiliations:** 1 Urban Health Collaborative, Drexel Dornsife School of Public Health, Philadelphia, PA USA; 2 Department of Epidemiology and Biostatistics, Drexel Dornsife School of Public Health, Philadelphia, PA USA; 3 Alberto Lleras Camargo School of Government, Universidad of the Andes, Bogotá, Colombia; 4 National Institute of Public Health, Cuernavaca, Mexico; 5 National Council for Science and Technology (CONACYT), Ciudad de Mexico, Mexico; 6 Escuela de Salud Pública, Universidad de Chile, Santiago de Chile, Chile; 7 CRONICAS Centre of Excellence in Chronic Diseases, Universidad Peruana Cayetano Heredia, Lima, Perú; 8 Observatory for Urban Health, School of Medicine, Federal University of Minas Gerais, Belo Horizonte, Brazil; 9 Institute for Information and Communication on Health – ICICT/FIOCRUZ, Rio de Janeiro, Brazil; 10 i3S - Instituto de Investigação e Inovação em Saúde, Porto, Portugal; 11 Departamento de Salud Pública, Pontificia Universidad Católica de Chile, Santiago de Chile, Chile; 12 Centro de Desarrollo Urbano Sostenible (CEDEUS), Santiago de Chile, Chile; 13 Instituto de Salud Colectiva, Universidad Nacional de Lanús, Buenos Aires, Argentina; 14 Department of City and Regional Planning, University of California Berkeley, Berkeley, CA USA; 15 CIECS - Centro de Investigaciones y Estudios sobre Cultura y Sociedad, Universidad Nacional de Córdoba, Cordoba, Argentina; 16 Universidad de São Paulo, São Paulo, Brazil; 17 Oswaldo Cruz Foundation, Salvador Bahia, Brazil; 18 Oswaldo Cruz Foundation, Rio de Janeiro, Brazil; 19 Universidade Federal de Minas Gerais, Belo Horizonte, Brazil; 20 School of Public Health, University of Chile, Santiago, Chile; 21 Department of Public Health, Pontificia Universidad Católica de Chile, Santiago, Chile; 22 Universidad de los Andes, Bogotá, Colombia; 23 Universidad de Ibagué, Ibagué, Colombia; 24 Universidad de Antioquia, Medellín, Colombia; 25 INCAP Research Center for the Prevention of Chronic Diseases (CIIPEC), Institute of Nutrition of Central America and Panama (INCAP), Guatemala City, Guatemala; 26 Instituto Nacional de Salud Pública, Cuernavaca, Mexico; 27 School of Medicine, Universidad Peruana Cayetano Heredia, Lima, Peru; 28 Brookings Institute, Washington, DC USA; 29 Department of City and Regional Planning, University of California Berkeley, Berkeley, CA USA; 30 Washington University in St Louis, St. Louis, MO USA; 31 Dornsife School of Public Health, Drexel University, Philadelphia, PA USA

**Keywords:** Public health, Epidemiology, Epidemiology

## Abstract

The concept of a so-called urban advantage in health ignores the possibility of heterogeneity in health outcomes across cities. Using a harmonized dataset from the SALURBAL project, we describe variability and predictors of life expectancy and proportionate mortality in 363 cities across nine Latin American countries. Life expectancy differed substantially across cities within the same country. Cause-specific mortality also varied across cities, with some causes of death (unintentional and violent injuries and deaths) showing large variation within countries, whereas other causes of death (communicable, maternal, neonatal and nutritional, cancer, cardiovascular disease and other noncommunicable diseases) varied substantially between countries. In multivariable mixed models, higher levels of education, water access and sanitation and less overcrowding were associated with longer life expectancy, a relatively lower proportion of communicable, maternal, neonatal and nutritional deaths and a higher proportion of deaths from cancer, cardiovascular disease and other noncommunicable diseases. These results highlight considerable heterogeneity in life expectancy and causes of death across cities of Latin America, revealing modifiable factors that could be amenable to urban policies aimed toward improving urban health in Latin America and more generally in other urban environments.

## Abstract (web summary)

City-level analysis of data from the SALURBAL project shows vast heterogeneity in life expectancy across cities within the same country, in addition to substantive differences in causes of death among nine Latin American countries, revealing modifiable factors that could be leveraged by municipal-level policies aimed toward improving health in urban environments.

## Main

Over 55% of the world’s population lives in urban areas and this number is expected to reach almost 70% by 2050 (ref. ^1^), highlighting the importance of studying the drivers of health in cities^2^. Even areas of the world that were traditionally more rural (such as parts of Asia and Africa) are rapidly becoming more urban^1^. In parallel with the growth of urban populations it has become increasingly clear that designing and managing cities in ways that protect the environment is key to achieving environmental sustainability. Moreover urban policies, such as the promotion of active transportation or a shift toward renewable energies^3^, can have considerable environmental and health co-benefits^4^, which support a case for identifying the features of cities that are associated with better population health and that can be modified with appropriate policies^5,6^.

Despite growing urban populations, worldwide research on the health effects of urban living is sparse and often inconsistent^7^. Studies comparing urban to rural areas in different countries can be difficult to interpret due to variations in definitions of urbanicity across countries^8,9^. In addition, many of these studies do not identify specific features of urban living that are damaging or enhancing to health. Although many studies have documented differences in life expectancy and cause-specific mortality across countries^10–13^ and, less frequently, across regions within countries^14,15^, few have investigated variations in health across cities and the factors associated with population health differences in these settings^16,17^. To our knowledge, no previous study has explored the heterogeneity in mortality and life expectancy outcomes in cities across multiple countries using comparable data.

City governments can directly affect population health in their municipalities, including mortality and environmental quality through implementation of appropriate urban policies. International organizations, such as UN-Habitat are increasingly calling for city-based and local initiatives, as well as recognition of cities as innovative, flexible and often progressive sites for policy-making to achieve the Sustainable Development Goals^2,9^; however, these actions must be based on evidence regarding what city-level factors are most important to health. Understanding the health consequences of urban growth, urban landscape features (such as urban extent fragmentation) and city social features (such as access to water) can help inform urban policies to promote health^9^.

As one of the most urbanized regions of the world and with many diverse cities^9^, Latin America provides a unique setting in which to investigate differences in life expectancy and causes of death across cities, both within and between countries. Such analyses can offer insights into the drivers of differences in population health across cities and how urban policies might be used to enhance health in cities globally. We used data compiled by the Salud Urbana en América Latina (SALURBAL) study^8,9^ to: (1) describe the variability in life expectancy and mortality profiles across Latin American cities in the period 2010–2016 and (2) investigate associations of city characteristics and life expectancy and mortality profiles.

## Results

### Heterogeneity in life expectancy across Latin American cities

**Fig. 1:**
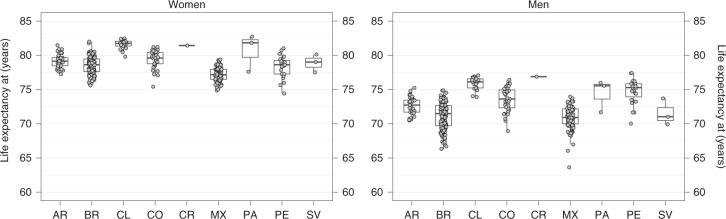
Variability in life expectancy at birth in 363 Latin American cities by country. Central line represents the median (50th percentile) city life expectancy, box limits represent the 25th and 75th percentiles and whiskers represent 1.5× the extent of the interquartile range. AR, Argentina; BR, Brazil; CL, Chile; CO, Colombia; CR, Costa Rica; MX, Mexico; PA, Panama; PE, Peru; SV, El Salvador. Source data

**Fig. 2:**
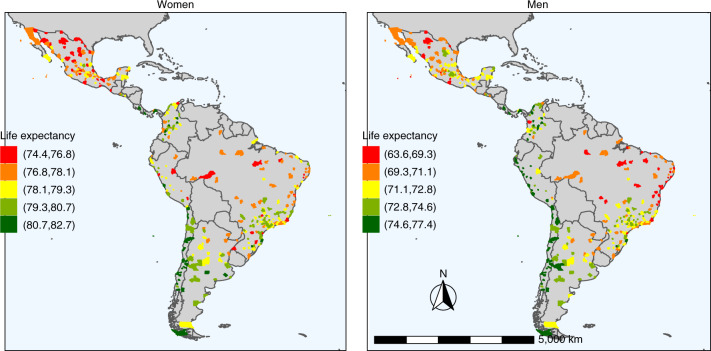
Spatial distribution of life expectancy at birth by city in 363 Latin American cities. The maps show life expectancy at birth for women (left) and men (right) in each city. The ranges (keys) are different for women and men. Source data

**Extended Data Fig. 1:**
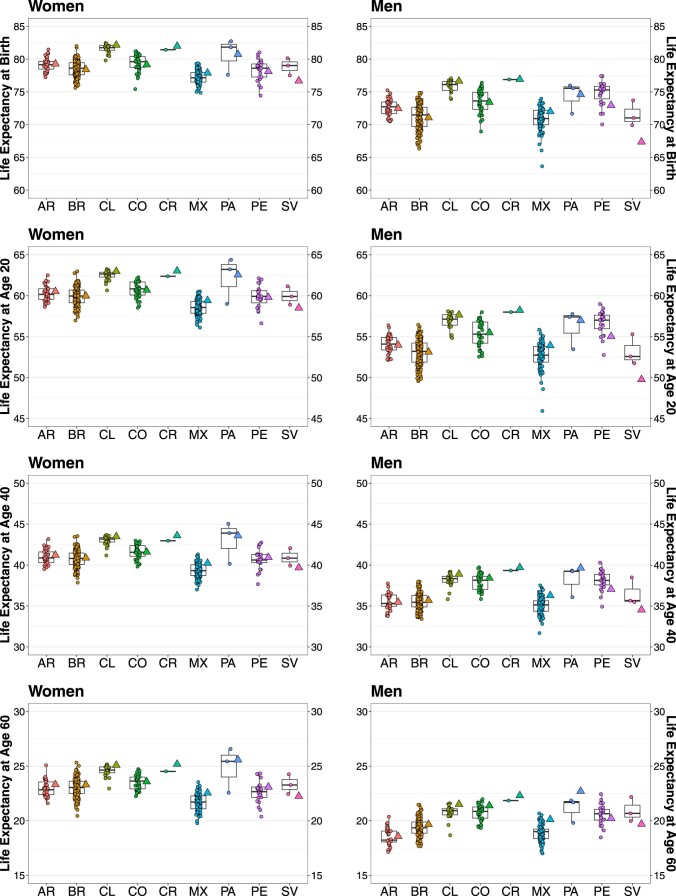
Variability in Life Expectancy at Birth, Ages 20, 40 and 60, in 363 Latin American cities by Country. Solid triangles represent the life expectancy at the country level for 2012–2016 (2010–2014 for El Salvador), as obtained from UNDP’s World Population Prospects (2019 version). The boxplot’s central line represents the median (50^th^ percentile) city life expectancy, box limits represent the 25^th^ and 75^th^ percentiles, and whiskers represent 1.5 times the extent of the interquartile range. AR: Argentina, BR: Brazil, CL: Chile, CO: Colombia, CR: Costa Rica, MX: Mexico, PA: Panama, PE: Peru, SV: El Salvador. Source data

Figures 1 and 2 show the distribution of life expectancy at birth in each of the 363 cities for men and women (Extended Data Fig. 1 shows the distribution of life expectancy for men and women at 20, 40 and 60 years of age). There was considerable heterogeneity in life expectancy across cities within each country, even when uncertainty in life expectancy estimation was accounted for: 41% and 46% of the total variability in life expectancy was within countries, in women and men, respectively (Supplementary Table 1). In men, the proportion of total variability that was within countries was higher for life expectancy at birth but was lower for life expectancy at ages 40 and 60 years. In women the proportion of the variability in life expectancy within countries did not vary as much across age and high variability persisted at ages 20, 40 and 60 years (Supplementary Table 1). We found that life expectancy at all ages, for both sexes and for all cities, was highly reliable with a relative s.e. (r.s.e. = s.e. / median life expectancy) <5% in all cases and <3% in the case of life expectancy at birth (Supplementary Fig. 1).

**Extended Data Fig. 2:**
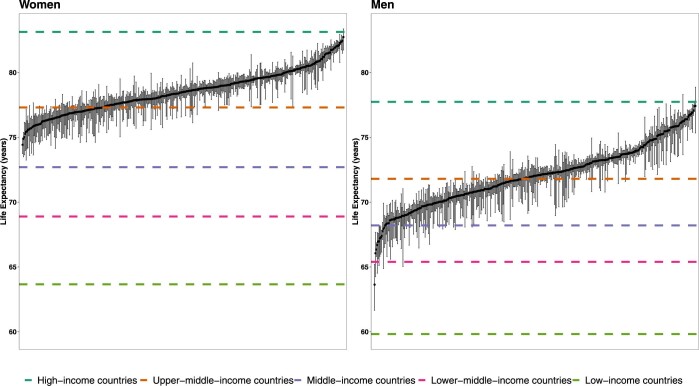
Life expectancy at birth in 363 Latin American cities and reference lines for 2012–2016 life expectancy by income group. Dashed lines represent life expectancy for World Bank income groups for 2012–2016, obtained from UNDP’s World Population Prospects (2019 version). Points represent life expectancy at birth in each city and error bars represent the 95% credible intervals of life expectancy at birth in each city. Source data

We found an 8- and 14-year range in life expectancy among the 363 cities, with the lowest values at 74.4 (95% credible interval (CrI) 73.7 to 74.9) and 63.5 (95% CrI 61.7 to 65.2) and the highest values at 82.7 (95% CrI 81.7 to 83.4) and 77.4 (95% CrI 75.3 to 78.9), in women and men, respectively (Figs. 1 and 2; city-specific data are available via the online interactive app https://drexel-uhc.shinyapps.io/MS10/). Extended Data Fig. 2 compares the life expectancy of each city with the life expectancy by income group for the 2012–2016 period, obtained from the 2019 United Nations Development Programme (UNDP) World Population Prospects^18^. For women, city life expectancy ranged from 74.4 to 82.7 years, which is slightly above that in middle-income countries (72.7 years) and slightly below that in high-income countries (83.1 years). Larger variation was observed for men, with city life expectancy ranging from 63.5 to 77.4 years, which is below that in lower-middle-income countries (65.4 years) and slightly below that of high-income countries (77.7 years).

Within countries, the largest ranges in life expectancy were observed in Brazil and Peru for women (ranges of 6.4 years (75.6 to 82.0) and 6.6 years (74.4 to 81.0), respectively) and in Brazil and Mexico for men (ranges of 8.6 years (66.3 to 74.9) and 10.4 years (63.6 to 74.0 years), respectively). Life expectancy also varied between countries, with Panama, Chile and Costa Rica having the cities with the longest life expectancy (81–82 years for women and 75–77 years for men). Mexico, Brazil and Peru had cities with the shortest life expectancy on average for women, at 77–78 years. Mexico and Brazil also had the shortest life expectancies for men, along with El Salvador, at 71 years on average. Figure 2 shows detailed spatial patterns of life expectancy in Latin America, with a North–South pattern in Brazil and Argentina and a coast-to-jungle pattern in Peru.

### Predictors of life expectancy in Latin American cities

**Extended Data Fig. 3:**
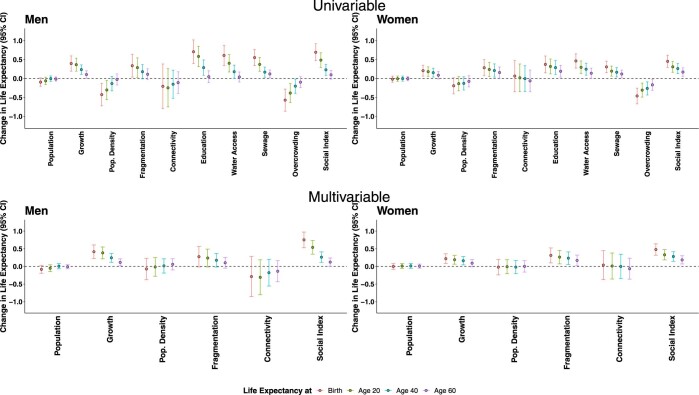
Results of the multivariable model with life expectancy at birth, 20, 40 and 60 years of age in 363 Latin American cities. Shown are regression coefficients and 95% confidence intervals. Horizontal dashed line represents the null effect. Source data

**Extended Data Fig. 4:**
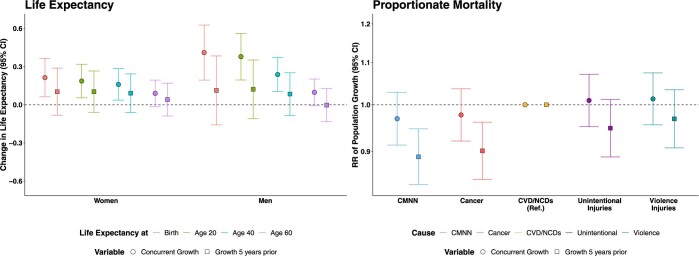
Results of secondary analysis using city growth in the 5 years previous to study period (instead of concurrent), in 363 Latin American cities. Shown are regression coefficients and 95% confidence intervals. Horizontal dashed line represents the null effect. Source data

Table 1 and Extended Data Fig. 3 show associations of urban characteristics with life expectancy at birth in multivariable models, scaled using s.d. to make coefficients comparable. Adjusted for other city-level indicators, the social environment index (a composite index of four indicators of area-level educational attainment, water and sewage access and overcrowding) predictive of longer life expectancy. Specifically, a 1 × s.d. higher social environment index was associated with a 0.78 and 0.48-year longer life expectancy in men and women, respectively. Moreover, we found that living in larger cities was associated with slightly shorter life expectancy among men and living in more fragmented cities (cities with a higher fragmentation of their urban extent, measured using the density of distinct urban patches) was associated with a longer life expectancy among both men and women. We also found that men living in cities with rapid population growth had longer life expectancy, but this association was not robust to the choice of the time period of population growth (Extended Data Fig. 4). Extended Data Fig. 3 shows the same set of associations for life expectancy at ages 20, 40 and 60 years, showing weaker associations especially in men. Extended Data Fig. 3 also shows unadjusted associations, where we found that the four separate components of the social environment index are predictive of life expectancy.

**Table 1:** Associations of city characteristics with life expectancy at birth among men and women in 363 Latin American cities

Variable^a^	s.d.	Men	Women
City size^b^	50% larger city^b^	−0.09 (−0.2;0.03)	0.00 (−0.08;0.08)
City growth	2.7% growth/5 years	0.43 (0.23;0.62)	0.22 (0.08;0.36)
Population density	4,145 population km^−^^2^	−0.09 (−0.39;0.22)	−0.02 (−0.24;0.20)
Fragmentation	0.3 urban patches km^−2^	0.28 (−0.02;0.58)	0.31 (0.10;0.53)
Street connectivity	6.4 intersections km^−^^2^	−0.27 (−0.85;0.31)	0.05 (−0.36;0.46)
Social environment index	1 × s.d.	0.78 (0.55;1.00)	0.48 (0.32;0.64)

Coefficients are differences in life expectancy (95% confidence intervals (CIs)), obtained from a linear mixed model of life expectancy, adjusted for the percentage of the administrative area of the city that is urbanized (Methods), with a random intercept for country, with all variables included in the same model. ^a^Variables are defined in Methods and supplementary information. All variables are scaled by their s.d., so their coefficient is interpreted as the difference in life expectancy per s.d. increase in the variable. ^b^City size was log transformed and its coefficients are interpreted as the increase in life expectancy in cities that are 50% larger.

### Heterogeneity in mortality profiles across Latin American cities

**Fig. 3:**
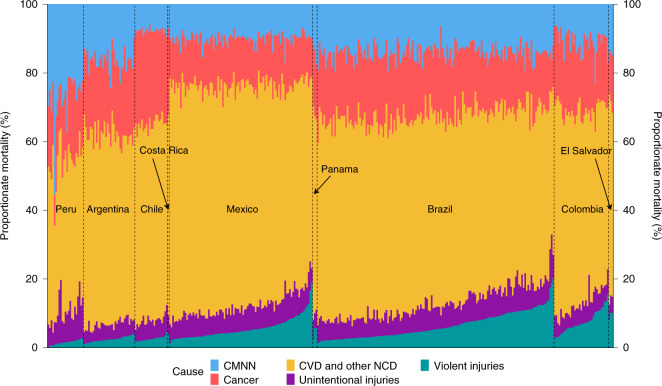
Variability in proportionate mortality in 363 Latin American cities by country. Each column is a city, with each color representing the proportion of deaths due to each cause. Cities are grouped into countries. Countries are sorted by the overall proportion of violent deaths in the country and cities are sorted within country by the proportion of violent deaths in each city. Source data

**Extended Data Fig. 5:**
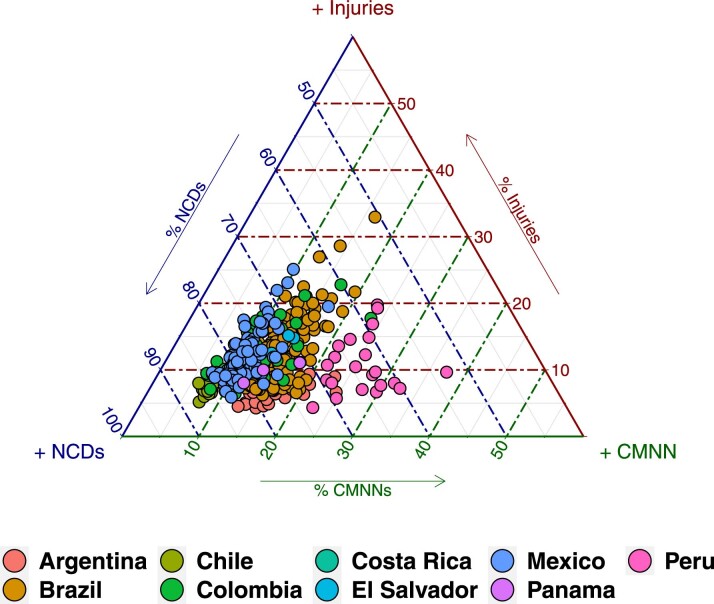
Variability in Proportionate Mortality in 363 Latin American Cities. Each dot represents a city. Source data

**Extended Data Fig. 6:**
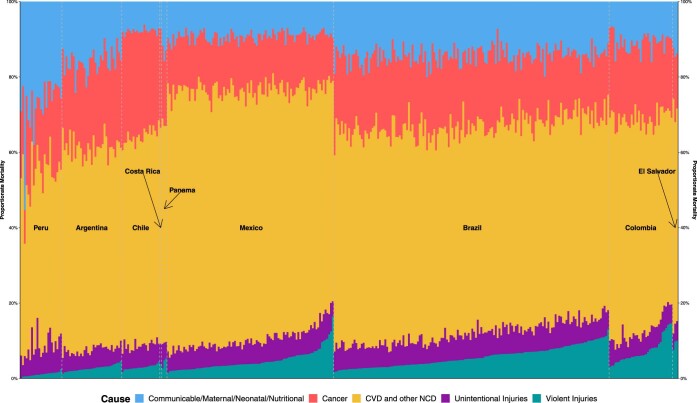
Age-adjusted proportionate mortality by city in 363 cities of 9 Latin American countries. Each column is a city, with each color representing the proportion of deaths due to each cause. Cities are grouped in countries. Countries are sorted by the overall proportion of violent deaths in the country, and cities are sorted within country by the proportion of violent deaths in each city. CVD and other NCDs: cardiovascular diseases and other non-communicable diseases. Source data

There was considerable variability in proportionate mortality deaths by a specific cause over total deaths by cause across cities (Fig. 3). Proportionate mortality (the proportion of all deaths that is due to a given group of causes) by communicable, maternal, neonatal and nutritional (CMNN) conditions varied from 6% to 55%, proportionate mortality by cancer varied from 9% to 30%, proportionate mortality by cardiovascular diseases (CVDs) and other noncommunicable diseases (NCDs) varied from 28% to 71%, proportionate mortality by unintentional injuries varied from 3% to 19% and proportionate mortality by violent injuries varied from 0% to 20% (Fig. 3, Extended Data Fig. 5 and Supplementary Table 2). CMNN, cancer and CVD/NCDs deaths varied more between countries than within countries (Supplementary Table 3; intraclass correlation coefficient (ICC) = 80%, 71% and 64%), with Peruvian, Chilean and Mexican cities having a higher proportionate mortality by CMNN, cancer and CVD/NCDs, respectively, compared with other countries. However, injury deaths showed large within-country variability (ICC = 38% for both unintentional and violent injuries), with cities ranging from 0% to 20% proportionate mortality by violent injuries in Mexico, Colombia and Brazil and from 4% to 19% proportionate mortality by unintentional injuries in Peru and Brazil. Variability in age-adjusted proportionate mortality (AAPM) was similar (Extended Data Fig. 6), although within-country variability in violent deaths was reduced (ICC = 48%, compared to 38% without age-adjustment; Supplementary Table 3), whereas within-country variability in unintentional injuries deaths was increased (ICC = 24% compared to 38%).

**Extended Data Fig. 7:**
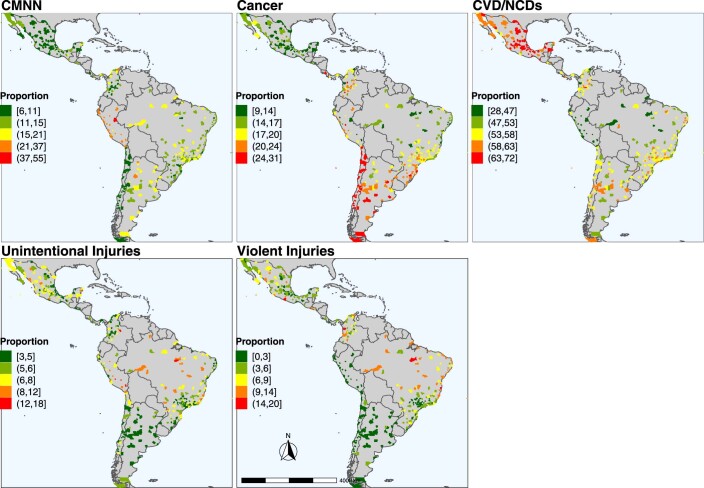
Spatial distribution of proportionate mortality in 363 Latin American cities. Source data

Extended Data Fig. 7 shows the spatial distribution of proportionate mortality. CMNN, while highest in Peru overall, had its highest levels specifically in cities of the Peruvian jungle. Argentina, with relatively high levels of proportionate mortality by CMNN, had a higher proportionate mortality in the northwestern cities of the country. Proportionate mortality by cancer, highest in Chile, was also high in Southern Brazil and in the Argentinian Pampas. CVD/NCDs were highest in Mexico, specifically in the center and northeastern parts of the country. Unintentional injuries were relatively high in cities in the Peruvian mountains and in North and Central Brazil, whereas violent injuries were highest in North and Northeastern Brazil, Southern Mexico and the Pacific coast of Colombia.

### Predictors of cause-specific proportionate mortality in Latin American cities

**Fig. 4:**
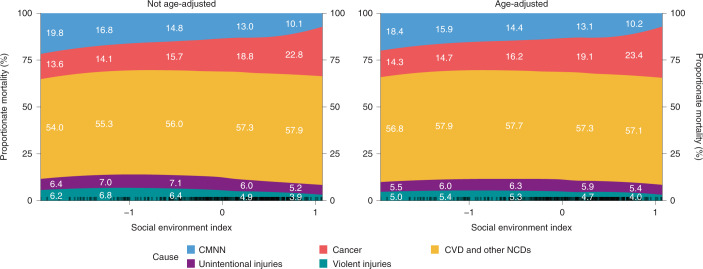
Social environment index and proportionate mortality by five causes in 363 Latin American cities. The range of the horizontal axis goes from the minimum to the maximum observed value of the social environment index. Ticks at the bottom of each plot represent observations (cities) with that value of the index. The white labels show proportionate mortality by each of the five causes by values of the social environment index (in five equal intervals).

**Extended Data Fig. 8:**
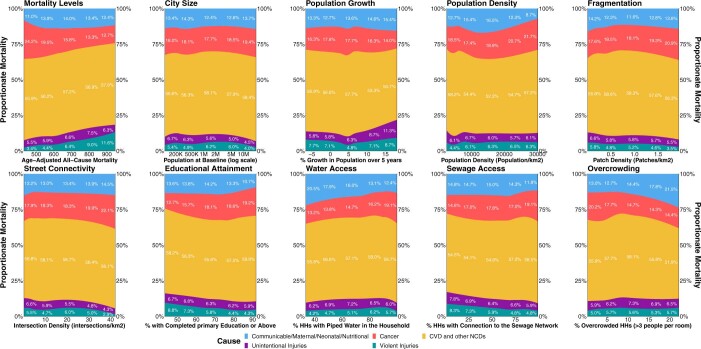
Predictors Proportionate Mortality by 5 causes in 363 Latin American Cities. The range of the horizontal axis goes from the minimum to the maximum observed value of each indicator. Ticks at the bottom of each plot represent observations (cities) with that value of each indicator. The white labels show proportionate mortality by each of the 5 causes by values of each indicator in 5 equal intervals. CVD and other NCDs: cardiovascular diseases and other non-communicable diseases.

**Extended Data Fig. 9:**
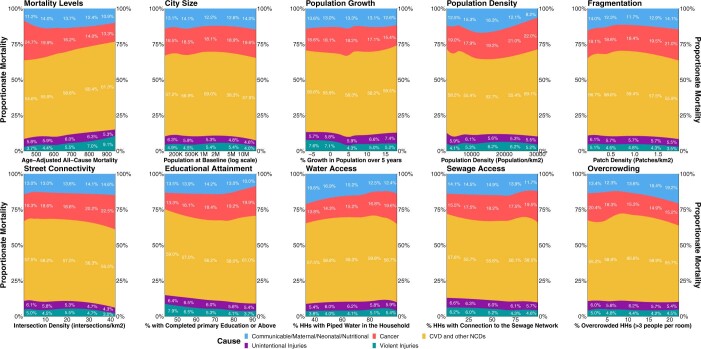
Predictors of Age-Adjusted Proportionate Mortality by 5 causes in 363 Latin American Cities. The range of the horizontal axis goes from the minimum to the maximum observed value of each indicator. Ticks at the bottom of each plot represent observations (cities) with that value of each indicator. The white labels show proportionate mortality by each of the 5 causes by values of each indicator in 5 equal intervals. CVD and other NCDs: cardiovascular diseases and other non-communicable diseases.

Higher attainment of the social environment indicators (lower overcrowding, higher water and sewage access and higher educational attainment) were associated with a lower CMNN and injury proportionate mortality and a higher cancer and CVD/NCDs proportionate mortality. Figure 4 shows the association of the social environment index with proportionate mortality (left) and AAPM (right): from the lowest to medium levels of social development, there is a sharp decrease in proportionate mortality by CMNN (20% to 15%) and an increase in CVD/NCDs (54% to 56%) and cancer (13% to 16%). From the medium to the highest levels, there is a further decrease in CMNN proportionate mortality (15% to 10%), an increase in cancer proportionate mortality (from 16% to 23%) and a decrease in unintentional injuries and violent injuries (from 7–8% to 4–5%). The pattern with AAPM was similar. Associations with higher educational attainment, water and sewage access and lower overcrowding were similar to the composite social environment index (Extended Data Figs. 8 and 9). Specifically, overcrowding had a very strong and steep association with CMNN, with proportionate mortality by CMNN around 13% at the lowest levels of overcrowding and 21% at the highest levels.

Extended Data Figs. 8 and 9 also show proportionate mortality and AAPM, respectively, by other city-level factors. Cities with a higher age-adjusted mortality rate have a higher violent injury proportionate mortality and a lower cancer proportionate mortality. Larger cities have a higher violent injury proportionate mortality, although this association was not observed for the largest cities in our sample. Cities with rapid growth have a higher CMNN and injury proportionate mortality and a lower cancer and CVD/NCDs proportionate mortality. Denser cities have a higher violent injury proportionate mortality and CMNNs first increase but then decrease with density, whereas cities with a less-connected street network and a more fragmented urban extent have a lower CMNN and higher CVD/NCDs proportionate mortality.

**Extended Data Fig. 10:**
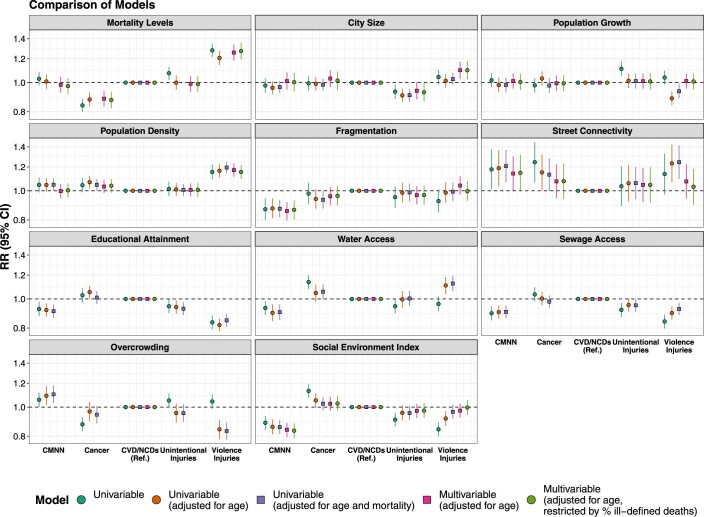
Rate Ratio for each group of causes of death (compared to CVD/Other NCDs) associated with a 1SD increase in city-level factors for six different levels of adjustment in 363 Latin American cities. Coefficients are Rate Ratios (As compared to CVD/NCDs), and error bars are their corresponding and 95% CIs, per standard deviation increase in each variable (except for city size, where they correspond to a 50% larger city). Univariable model contains each variable in a different model, adjusted for % built-up (see methods). Univariable models adjusted for age contain a covariable for % under 15 and % above 65, and univariable models adjusted for mortality contain a covariable for all-cause age-adjusted mortality rate. Multivariable models contain all predictors in the same model. The model restricted by % ill-defined deaths restricts the analysis to cities with <13% ill-defined deaths (90^th^ percentile). Source data

Table 2 shows associations of each city-level predictor with proportionate mortality for each cause relative to CVD/NCD, adjusted for all other predictors and age (Extended Data Fig. 10 shows a comparison for different sets of adjustments). Cities with higher all-cause age-adjusted mortality have relatively higher proportionate mortality due to violent injury, whereas larger cities have higher violent injury proportionate mortality and lower unintentional injury proportionate mortality. Dense cities had more violent deaths and less-fragmented and more-connected cities, along with cities with lower values of the social environment index, had more CMNN deaths, all relative to CVD/NCD deaths. While population growth was not associated with proportionate mortality, in sensitivity analysis using population growth in the 5 years before the study time (rather than concurrent with the study time), we found that population growth was associated with lower CMNN and cancer proportionate mortality (Extended Data Fig. 4). Sensitivity analysis excluding cities with the highest proportion of ill-defined deaths showed no difference in results (Extended Data Fig. 10).

**Table 2:** Rate ratio (95%) for each group of causes of death (compared to CVD/other NCDs) associated with a 1 × s.d. increase in city-level factors, adjusted for all other city-level factors, the percentage of the administrative area of the city that is urbanized and the percentage of the population aged 15 years or below and 65 years or above

Variable	s.d.^a^	CMNN	Cancer	CVD/NCDs (reference group)	Unintentional	Violent
Mortality^b^	94.3 deaths per 100,000	0.99 (0.93;1.05)	**0.88** (**0.83;0.94)**	1.00	1.01 (0.95;1.07)	**1.27** (**1.20;1.35)**
City size^c^	50% larger city^c^	1.01 (0.95;1.08)	1.03 (0.97;1.10)	1.00	0.95 (0.89;1.01)	**1.10** (**1.03;1.18)**
City growth^b^	2.7% growth in 5 years	1.01 (0.96;1.07)	0.99 (0.94;1.05)	1.00	1.02 (0.96;1.08)	1.02 (0.96;1.08)
Population density^b^	4,145 population km^−2^	1.00 (0.95;1.05)	1.03 (0.98;1.09)	1.00	1.02 (0.97;1.07)	**1.17** (**1.11;1.23)**
Fragmentation^b^	0.3 urban patches km^−2^	**0.86** (**0.80;0.92)**	0.96 (0.89;1.02)	1.00	0.96 (0.90;1.03)	1.04 (0.97;1.11)
Street connectivity^b^	6.4 intersections km^−2^	**1.14** (**1.00;1.30)**	1.07 (0.95;1.22)	1.00	1.04 (0.91;1.18)	1.06 (0.93;1.21)
Social environment index	1 × s.d.	**0.84** (**0.80;0.89)**	1.03 (0.97;1.08)	1.00	0.97 (0.92;1.02)	0.97 (0.92;1.02)

All variables are included in the same model. This analysis includes 362 cities, as Zarate-Campana in Argentina has no available street network data. ^b^Coefficients are rate ratios (as compared to CVD/NCDs) and 95% CIs, per s.d. increase in the variable. ^c^Coefficients are rate ratios (as compared to CVD/NCDs) and 95% CIs, in a 50% larger city. ^a^Refers to the scale of the variable (for example, mortality coefficients are interpreted per 107.8 deaths/100,000 people increase or 1 × s.d. of age-adjusted mortality rate). Ref., reference group (CVD/NCDs).

## Discussion

Our findings demonstrate that life expectancy and mortality profiles are highly heterogeneous across Latin American cities. Life expectancy at birth ranges from 74–83 years and 63–77 years in women and men, respectively. While countries such as Panama, Costa Rica and Chile have higher levels of life expectancy, in many countries there is large variability in life expectancy across cities within each country, sometimes as large as a difference of 7–10 years, as is the case with Mexico, Brazil, Colombia and Peru. We also found that social environment variables were predictive of life expectancy. The heterogeneity in mortality profiles between and within countries varied widely by cause. While NCDs were the most common cause of death and injuries were the least common, the proportion of deaths by each cause varied substantially across cities. We found that CMNN, cancer and CVD/NCD deaths varied more between countries and unintentional injuries and violent injuries deaths varied more within countries. We also documented several important patterns regarding associations of city features with cause-specific mortality. We found that large and/or dense cities had a higher proportionate mortality by violent injuries and less-fragmented and more-connected cities had a higher proportionate mortality by CMNN. Cities with better social environment indicators had lower CMNN, whereas the association with CVD/NCDs and cancer varied over the range of social environment values.

The usual conceptualization of the urban advantage in health^7^ leaves an open question of whether health outcomes are similar across cities and whether there is a role for city-level factors as determinants of health^5^. We have shown that there is large variability in life expectancy and proportionate mortality, even when considering cities in the same country.

Few studies have examined life expectancy at the city level across different countries. Cities in our sample with the lowest life expectancy at birth for men (63 years in Acapulco, Mexico), had life expectancies similar to Botswana and Myanmar, whereas the lowest city life expectancies for women (74–75 years in Acapulco, Acuña and Juarez in Mexico, Riohacha in Colombia and Chimbote in Peru) were similar to the life expectancy of cities in Egypt and Bangladesh. Cities with the highest life expectancy for men (77 years in Ica and Lima in Peru, San Jose in Costa Rica and Talca, La Serena and Santiago in Chile) were similar to high-income countries such as Portugal and Slovenia, whereas the highest life expectancy for women (82–83 years in Los Angeles, Santiago, La Serena and Valparaiso in Chile and David in Panama) was similar to the United Kingdom and Germany (Fig. 2 shows a comparison with life expectancy by World Bank income groups, all for 2012–2016, obtained from the 2019 UNDP World Population Prospects)^18^.

With respect to mortality profiles, we observed heterogeneities across cities that differed by cause. For example, the range of proportionate mortality by violent injuries varies from close to 0%, similar to that of Italy or Greece, to around 20%, similar to rates in Iraq^13^. Mortality by CVD/NCDs ranged from 28%, similar to Togo or Madagascar, to 71%, similar to Serbia or Romania^13^. The Global Burden of Disease study looked at variations between states within both India^14^ and Mexico^15^, but has not done so at a city level. While states are meaningful political units, cities can have more pronounced effects on health and mortality profiles through local urban policies^19^. The rise of municipalism and the coordinated efforts of cities across the world, offers the opportunity to develop tailored policies at the local level that draw strength from the specific particularities of each city, but that also acknowledge their connections to other cities or parts of the world^20^.

We found that social environment indicators, including education, access to water and sanitation and overcrowding, were strongly associated with life expectancy. This association has two potential explanations^21^: that these indicators are markers of improved living conditions and health-promoting services relevant to multiple causes of death across multiple ages or that these indicators capture processes specific to certain causes of death. For example improved sanitation and access to water are linked to lower rates of diarrhea and other communicable diseases^22^, lower levels of overcrowding are linked to a lower rate of respiratory infections^23^ and increased area-level socioeconomic status is associated with lower cardiovascular disease mortality in US counties^24^. We found that these associations were weaker for life expectancy at ages 20, 40 and 60 years compared with life expectancy at birth, which is consistent with other literature from the region showing a lack of a social gradient in mortality for the elderly^25^.

Social environment variables also showed consistent associations with proportionate mortality. Higher levels of education attainment and water and sewage access and lower levels of overcrowding were associated with a higher proportion of cancer and CVD/NCDs and a lower proportion of CMNN. The higher proportion of NCDs in areas with improved markers of social environment is consistent with the epidemiological/demographic transition into stage 3 (ref. ^26^), the stage of social development associated with an increase in mortality related to NCDs, highlighting the need for better NCD control strategies in these cities^27^.

Overall, cities with a better social environment had longer life expectancy (and therefore, lower overall levels of mortality) and higher mortality by NCDs, consistent with epidemiologic transition models^26^. However, as has been noted^28^, later stages in the epidemiologic transition can also be characterized by large health inequities (which we have shown to be very wide in Latin America^29^) and by large heterogeneities in the proportion of deaths from injuries^28^. That we found no adjusted association of social environment indicators with violence mortality (and only a weak negative association with unintentional injuries) highlights that, at least for Latin American cities, injury mortality occurs at all levels of the social development spectrum.

We found that life expectancy was shorter for men living in large cities, but found no association for women. We also found that larger cities had a relatively higher proportionate mortality by violence compared with smaller cities. Given that in Latin America, violent deaths are strongly concentrated among young men, both results are consistent with a higher burden of violence in large cities. This is consistent with some previous research showing higher rates of violent crime in large cities both in the United States^30^ and in Brazil, Mexico and Colombia^31^. We also found that more fragmented cities had longer life expectancy and a relatively lower proportionate mortality by CMNN, whereas cities with more densely connected street networks had proportionately higher CMNN. This is consistent with previous research on the relationship of the fragmentation of urban ecosystems and infectious diseases^32,33^. Last, cities with high population density had a higher proportionate mortality by violent injuries, especially comparing those of medium density versus low density, similar to research in the United States that found that increases in density at the low end of the spectrum are associated with higher violence^34^.

This study has several strengths. First, we included 5 years of data on all deaths and population of all 363 cities above 100,000 people in nine Latin American countries, representing 283.3 million people (almost half of the entire population of the region of Latin America and the Caribbean). These countries represent a wide variety of social and economic conditions, from lower-middle to high-income countries^35^. This constitutes a comprehensive effort to characterize life expectancies and mortality profiles across the universe of cities of these countries in Latin America. Second, we have harmonized and standardized mortality records in each city^8^ and have tried to correct several issues that are present in analyses of vital registration records, including lack of complete coverage, using city-specific corrections. Third, we have also used categorizations of causes of death employed in previous studies, improving the generalizability of our results^8^. Last, we have examined these associations using robust multilevel models, both univariable and multivariable, including nonparametric models. Future studies using these data will examine how various specific factors are related to changes in life expectancy over time, including air pollution, measures of income equality or characteristics of the healthcare system.

This study has a number of limitations. First, while we corrected for potential under-reporting of deaths by estimating completeness at the city level, there is potential for this correction to not be enough or to be overcorrecting our mortality rates (increasing them spuriously). We implemented common demographic methods for the estimation of levels of coverage of mortality and used them in a way that maximizes adherence to assumptions^36^, especially the lack of migration assumption. Our analysis of proportionate mortality data should be robust to these biases, given that our estimates provide associations for the relative increase in certain causes of death compared to overall mortality, which should be robust to undercounting providing that there is consistency in the under-reporting by cause of death. Second, the quality of data regarding age may also be problematic, as there is reported age over-estimation in mortality records, age-underestimation in population estimations and age heaping^37^. Third, we rely on the causes of death as coded in death certificates, where some deaths are coded as ill-defined^38^; however, we redistributed these to other causes using a proportional simple imputation based on age, sex, country and year^38,39^. Our sensitivity analysis to test the robustness of our results to a high proportion of ill-defined deaths showed no changes to our main inferences. Another limitation is the different timing in the measurement of social environment measures, for which we rely on censuses conducted at different times in each country. Last, our analysis was conducted at the city level, so inferences about more granular levels (neighborhoods, households and individuals) are limited^40^.

There is large heterogeneity in life expectancy and mortality profiles across cities of Latin American countries. Population growth and social environment indicators are positively associated with life expectancy, whereas city size, growth and built and social environment features are associated with different causes of death. Characterizing heterogeneity of population health and factors that influence health across cities is the first step toward identifying risk factors that can be effectively modified by urban and health policies, as well as their implementation and continued monitoring for the region.

## Methods

### Study setting

We conducted this study as a part of the SALURBAL project^8,9^, which has compiled and harmonized health, social and physical environment data on all cities with a population above 100,000 in 11 Latin American countries (Argentina, Brazil, Chile, Colombia, Costa Rica, El Salvador, Guatemala, Mexico, Nicaragua, Panama and Peru). The SALURBAL study protocol was approved by the Drexel University Institutional Review Board (ID no. 1612005035).

Cities of 100,000 people or more in 2010 were identified by combining information from the Atlas of Urban Expansion, census-based population data on administratively defined cities in each country and inspection of built-up areas on satellite maps^8^ Using this approach, 371 cities were identified and operationalized as clusters of the smallest administrative units for which disaggregated vital statistics data were available. More details on city selection and definition are available elsewhere^8^.

For this study, we used data on 363 cities in nine countries for which mortality and population data were available at the city level: Argentina, Brazil, Chile, Colombia, Costa Rica, El Salvador, Mexico, Panama and Peru. We summed deaths and population counts for each city during a 5-year period (2012–2016, except for El Salvador, which was 2010–2014 owing to restricted data availability). Nicaragua and Guatemala were excluded because of a lack of mortality data with georeferenced location of residence.

### Data sources

We obtained mortality data from vital registration systems in each country. Mortality records included data on municipality of residence, age at death and cause of death coded using the International Classification of Diseases v.10. Population projections or estimations at the city level for every year from 2010 to 2016 by age were obtained from national census bureaus. Data on predictors were obtained from vital statistics, population projections, censuses (latest available for every country), the Global Urban Footprint Project (2012), Worldpop (2010) and OpenStreetmap (2017). More details are available elsewhere^8^ and in Supplementary Tables 4–6.

### Vital registration data

We addressed three challenges of vital registration. First, we imputed data with missing sex (~0.2%) and age (~0.5%) using a single conditional imputation. We imputed sex in each record with missing sex using the observed proportion of males in the same age (5-year groups) and cause of death strata for each country year. The same procedure was used for imputing age using sex and cause of death.

Second, we corrected for the lack of complete registration of all deaths at the city level, using an ensemble of death distribution methods^41–43^, stratified by sex. These methods estimate the degree of coverage of deaths using population by age at two points (coinciding with the study period) and deaths in the period in between. We estimated coverage using three different methods: generalized growth balance (GGB)^44^, synthetic extinct generations (SEG)^45^ and the hybrid method^41^. To address the assumption of lack of net migration, we used two strategies. First, we obtained the average of the coverage estimates, an approach that has been proposed to adequately account for migration flows to cities^36,46^. Specifically, we computed the harmonic mean of the GGB, SEG and hybrid methods^46^. Second, we calculated estimates of coverage for three age bands. First, we used the best-fitting age bands as provided by the DDM R package^47^, a method used before in Ecuador^46^. Second, we also used manually specified age bands, specifically the ages proposed by Hill^36^ (30–65 years) and Murray^43^ (50–70 years), as these are age ranges where migration is lower than at younger ages. The combination of three methods and three age bands resulted in nine estimates of completeness per city, which we incorporated in our models as detailed below. Supplementary Fig. 2 shows the distribution of estimates of completeness of death counts by city and country.

Third, we redistributed deaths assigned to ill-defined diseases or injuries to specific causes of death^38,39^. We did this redistribution in three steps. First, for every 5-year age group, sex, country and year, we obtained the observed distribution of causes of death, diseases (CMNN, cancer and CVD/NCDs) and injuries (unintentional and violent). Second, for all deaths by an ill-defined disease in each 5-year age group, sex, country and year, we assigned a cause of death (CMNN, cancer or CVD/NCDs) using a single multinomial draw, with the observed probabilities from step 1. For all deaths by an injury of ill-defined intent in each 5-year age group, sex, country and year, we assigned a cause of death (unintentional or intentional) using a single multinomial draw, with the observed probabilities from step 1. Supplementary Table 2 and Supplementary Fig. 3 show the proportion of deaths by ill-defined diseases and by injuries of ill-defined intent by city and country. Ill-defined diseases represented 3% of all deaths and ranged from 0 to 32% by city, although 90% of the cities had <13% ill-defined deaths. Ill-defined deaths were highest in Argentina, Brazil and El Salvador (6%, 4% and 15%, respectively). Injuries of ill-defined intent represented <0.1% of all deaths, ranged from 0 to 28%, although all but four cities had <5% of all deaths due to injuries of ill-defined intent.

### City-level predictors

We purposively selected nine predictors available in routine data sources and that also captured three dimensions potentially amenable to urban policies: demographic features, urban form characteristics and social environment characteristics. Demographic features included city size (population) and city growth (population growth in the 5 years of the study). Age-adjusted all-cause mortality rate (using the 2000–2025 World Health Organization standard population) was also included as a covariate in analyses of proportionate mortality by cause. To characterize urban form, we used population density (population per area), fragmentation of the urban extent (density of urban patches) and connectivity (density of street intersections). To characterize the social environment, we used the proportion of the population aged 25 years or above that completed primary education and the proportion of households that were overcrowded, had water in the dwelling and connection to the sewage network. We also created a social environment index as the sum of the *z* scores of education, water access, sewage access and overcrowding (reversed). A higher level of the social environment index is a proxy for improved social conditions. All features refer to the administrative extent of the city. Supplementary Tables 4 and 5 show detailed definitions, operationalization and data sources for all indicators and Supplementary Table 7 shows a description by city size.

### Estimation of life expectancy

Life expectancy is a widely used marker of the health of populations, as it summarizes mortality across ages and is a number that can be easily understood in terms of years of life^10^. To estimate life expectancy, we obtained estimates of age- and sex-specific mortality rates using a Bayesian Poisson model that draws information from country-level mortality patterns and age-specific mortality patterns. This model also incorporates uncertainty from the estimation of under-registration of death counts, following the approach of Schmertmann and Gonzaga^48^.

We let *y*_*i*(*j*)*k*_ and *n*_*i*(*j*)*k*_ denote the number of deaths in age group *k* from city *j* of country *i*, where *k* = 1,…17, *i* = 1,…9, *j* = 1,…*J*_*i*_, and *J*_*i*_ denotes the number of cities in our dataset that belong to country *i*. To model these death counts, we assume

1 $$y_{i\left( j \right)ks}\sim Pois\left( {c_{i\left( j \right)s}n_{i\left( j \right)ks}\lambda _{i\left( j \right)ks}} \right)$$

where *λ*_*i*(*j*)*ks*_ denotes the city/age/sex-specific mortality rate and *c*_*i*(*j*)*s*_ is a correction factor that measures the estimated proportion of deaths observed for each city and sex. Due to the potential for small counts in our data, we made use of Bayesian models to produce more precise estimates of the *λ*_*i*(*j*)*ks*_. First and foremost, we write

2 $$\log \lambda _{i\left( j \right)ks} = \beta _{0ks} + \alpha _{js} + z_{i\left( j \right)ks}$$

where *β*_0*ks*_ denotes an age/sex-specific intercept term, *α*_*js*_ denotes a country-specific random effect and *z*_*i*(*j*)*ks*_ is a random effect that permits the flexibility for each city to have its own trends in mortality across age groups. While sufficient data exist to produce stable estimates of the *β*_0__*k*_ parameters without informative prior structures, estimating the remaining parameters may require additional considerations. For instance, some countries have very few cities (for example, Costa Rica has only one city included in our study, Panama and El Salvador have three cities each); thus, to prevent overfitting we shrink the *α*_*js*_ toward each other by assuming $\alpha _{js}\sim Norm\left( {0,\tau _s^2} \right)$, where $\tau _s^2$ controls the degree of shrinkage. To model the city-specific random effects, however, we require a more nuanced approach. In particular, while we want to let *z*_*i*(*j*)*ks*_ vary by both city and age, the mortality schedules by age suggest that a simple linear trend in age would be inappropriate. However, we would also like to avoid sudden sharp increases or decreases in the *z*_*i*(*j*)*ks*_ across age, instead favoring more smooth/gradual changes in age-specific mortality rates at the city level. Thus, we consider the use of an autoregressive structure for each city’s *z*_*i*(*j*)*ks*_ where we assume

3 $$z_{i\left( j \right)1s}|\sigma _{1s}^2\sim Norm(0,\sigma _{1s}^2){\mathrm{for}}\,{\mathrm{age}} - {\mathrm{group}}\,0 - 1$$

4 $$z_{i\left( j \right)ks}|z_{i\left( j \right),k - 1,s}\sigma _k^2\sim Norm(\rho _sz_{i\left( j \right),k - 1,s},\sigma _{ks}^2){\mathrm{for}}\,{\mathrm{age}} - {\mathrm{groups}} > 0 - 1$$

where *ρ*_*s*_ denotes a between-age correlation parameter and the $\sigma _{ks}^2$ parameters facilitate between-city shrinkage in the *z*_*i*(*j*)*ks*_. Thus in our modeling approach, the city-specific mortality rate for a given age group is assumed to be centered around a value based on which country it belongs to ($\exp \left[ {\beta _{0ks} + \alpha _{js}} \right]$) with deviations from this trend being permitted when strong evidence exists (for example, greater than expected death counts among consecutive age groups).

We account for potential undercounting by following the approach of Schmertmann and Gonzaga^48,49^. We begin by calculating the nine completeness estimates described above (GGB, SEG and the hybrid GGB–SEG, by the three potential age bands, automatic, 30–65 and 50–70), which we denote $c_{i\left( j \right)s;f}^ \ast$ for $f = \left\{ 1, \ldots ,9 \right\}$. We then let *ϕ*_*i*(*j*)*s*_ denote the harmonic mean (as suggested by Peralta^46^),

$$\phi _{i\left( j \right)s} = \left( {\mathop {\sum}\limits_{\mathrm{f}} {\frac{1}{{c_{i\left( j \right)s;f}^ \ast }}} } \right)^{ - 1}\,{\mathrm{and}}\,K_{i\left( j \right)s} = \frac{{\phi _{i\left( j \right)s}\left( {1 - \phi _{i\left( j \right)s}} \right)}}{{s_{i\left( j \right)s}^2}} - 1$$

where

$$s_{i\left( j \right)s}^2 = \mathop {\sum}\limits_f {\left( {c_{i\left( j \right)s;f}^ \ast - \phi _{i\left( j \right)s}} \right)} /\left( {9 - 1} \right)$$

represents the sample variance of the $c_{i\left( j \right)s;f}^ \ast$ correction factors relative to their harmonic mean. We then let the correction factor in our model, *c*_*i*(*j*)_*s*, have a prior distribution of the form:

$c_{i\left( j \right)s}\sim Beta\left( {K_{i\left( j \right)s}\phi _{i\left( j \right)s},K_{i\left( j \right)s}\left( {1 - \phi _{i\left( j \right)s}} \right)} \right),$ which yields a prior whose expected value is *ϕ*_*i*(*j*)__*s*_ and variance is $s_{i\left( j \right)s}^2$, as desired. It should be noted that our goal is not necessarily to learn about the *c*_*i*(*j*)_*s* but rather to account for the uncertainty in our estimate of the true proportion of undercounting in the data.

This model was fitted using iterative Markov chain Monte Carlo (MCMC) using the JAGS program. The estimation for the model starts with 50,000 burn-in MCMC iterations that allow the chain to converge, followed by 100,000 MCMC iterations. To reduce autocorrelation we thinned these samples by a factor of 100 to obtain 1,000 complete sets. We obtained a sample of 1,000 complete sets of age- and sex-specific mortality rates (λ) from these iterations. From this model, we obtained a complete set of 1,000 age- and sex-specific mortality rates that were used henceforth. Finally, we calculated a set of 1,000 life expectancies for each city and sex using life tables, both at birth and at ages 20, 40 and 60 years, using the DemoTools R package^50^ (lt_abridged function).

### Mortality profiles

Mortality profiles by cause of death were operationalized using cause-specific proportionate mortality, estimated as number of deaths in a specific cause divided by the total number of deaths:

$${\rm{PM}}_{ij} = \frac{{{\rm{Deaths}}_{ij}}}{{\mathop {\sum }\nolimits_{i = 1}^I {\rm{Deaths}}_{ij}}}$$

Where PM_*ij*_ is the proportionate mortality for the *i*th cause in the *j*th city and deaths_*ij*_ are the number of deaths due to the *i*th cause in the *j*th city. Deaths were categorized using the Global Health Estimates classification^51^ and then grouped into five categories: (1) CMNN; (2) cancer; (3) CVD/NCDs; (4) unintentional injuries; and (5) violent injuries. Supplementary Table 8 contains a comprehensive list of International Classification of Diseases codes included in each category. In secondary analyses, we also calculated an AAPM, by using cause-specific age-adjusted mortality rates (using the World Health Organization 2000–2025 population):

$${\mathrm{AAPM}}_{ij} = \frac{{{\mathrm{Age}}{\hbox{-}}{\mathrm{adjusted}}\,{\mathrm{mortality}}\,{\mathrm{rate}}_{ij}}}{{\mathop {\sum }\nolimits_{i = 1}^I {\mathrm{Age}}{\hbox{-}}{\mathrm{adjusted}}\,{\mathrm{mortality}}\,{\mathrm{rate}}_{ij}}}$$

In descriptive analysis, to account for the lack of complete registration of all deaths, we upweighted deaths by the correction factor (corrected deaths = observed deaths / coverage). For the multilevel models, we downweighed population by the correction factor to avoid increasing the precision of our estimates artificially (corrected population = observed population × coverage).

### Statistical analysis

The main objectives of this analysis were to describe heterogeneity in life expectancy and mortality profiles between Latin American cities and to estimate associations with city-level factors.

To describe variability in life expectancy, we created graphical depictions of life expectancy by city and country, showing the median and (where appropriate) 95% CrIs of life expectancy for each city. We also calculated and plotted the r.s.e., a measure of the sampling variability of life expectancy. We considered an estimate reliable if r.s.e. < 25%^52^.

We also decomposed the variability in life expectancy into: (1) differences between the 1,000 iterations within each city; (2) differences between cities within a country; and (3) differences between countries using a three-level linear mixed model with life expectancy as the dependent variable and a random intercept for country and city:

$$\begin{array}{l}{{{\rm{Life}}\,{\rm{Expectancy}}}}_{ijk} = \alpha _{000} + \mu _{00k} + \mu _{0jk} + {\it{\epsilon }}_{ijk}\\ \mu _{00k}\sim N\left( {0,\tau _{000}} \right);\mu _{0jk}\sim N\left( {0,\tau _{00}} \right);{\it{\epsilon }}_{ijk}\sim N\left( {0,\sigma ^2} \right);\end{array}$$

Where life expectancy_*ijk*_ is the life expectancy for each *i*th iteration in the *j*th city in the *k*th country, *α*_*000*_ is the overall mean life expectancy, *μ*_*00k*_ is the deviation of each country mean from the overall mean, *μ*_*0jk*_ is the deviation of each city’s mean from the country mean and *ε*_*ijk*_ is the deviation for each iteration from the city mean. These three random effects (country random intercept, city random intercept and iteration residuals) are distributed normally with variances *τ*_*000*_, *τ*_*00*_ and *σ*^2^, respectively. We calculated how much of the total variance (*τ*_*000*_ + *τ*_*0*0_ + *σ*^2^) was at the iteration level (*σ*^2 ^/ total variance), city level (*τ*_00 _/ total variance) and country level (*τ*_*000*_ / total variance). The linear mixed model to decompose variance was weighted by the population of each city at baseline. We also computed ranges (max–min) for life expectancy for each country, age and sex combination, to assess the variability for specific countries.

To describe heterogeneity in mortality profiles across cities we created graphical depictions of proportionate mortality and computed ICCs to describe between- versus within-country variability, using a linear mixed model with each proportionate mortality as the outcome and a random intercept for country.

To estimate the univariable association of city-level predictors with life expectancy, we ran set of a linear mixed models with life expectancy as the outcome, a random intercept for country and a single predictor in each model. This model provides a descriptive look at the variation in life expectancy by levels of city size, physical environment and social environment variables. The linear mixed model was run 1,000 times (with the 1,000 estimated life expectancies for each city) and then coefficients were pooled using Rubin’s formula^53^. We also ran a multivariable model with all urban form variables and the social environment index. This model provides a description of the variation in life expectancy by levels of each predictor, adjusted for all other predictors.

We examined the association of city-level factors with proportionate mortality using two approaches. First, we used a nonparametric approach to describe the association of each city-level factor with proportionate mortality by each group of causes of death. We computed a LOWESS smoother of each proportionate mortality on the city-level factor. We then created stacked area plots showing the estimated levels of proportionate mortality for the range of levels of the city-level predictor.

Second, to provide an estimate of the strength of these associations, we fitted a three-level negative binomial multilevel model for aggregated data, where each observation was a cause of death–city–country combination, with an offset for log(population), a random intercept for city and country. The model includes a set of four indicator variables for cause of death, the variable of interest and an interaction term between causes of death and the exposure. The exponentiated interaction coefficients represent the relative increase in the proportion of deaths by a specific cause as compared to CVD/NCDs per one-unit increase in the predictor. We fitted these models at different adjustment levels: (1) a model with each predictor investigated separately (univariable model); (2) a univariable model + adjustment for the percentage of the population under 15 and above 65 (age-adjusted univariable model); (3) a univariable model adjusted for age and all-cause mortality, to evaluate the sensitivity of the estimates to heterogeneity in the absolute levels of mortality rates; and (4) a multivariable model with all predictors included in the same model, along with age. In the multivariable models we included only the social environment index instead of including all social environment indicators to avoid collinearity between social environment indicators.

In all models above, and to make coefficients comparable, all variables were centered by their overall mean scaled by their overall s.d., except for population, which was log transformed. All analyses that used built environment variables (density, fragmentation, connectivity) were adjusted for the proportion of the administrative area that is urbanized (covered by urban patches^8^), to avoid measurement misclassification of built environment variables due to the definition of the administrative area and to standardize cities by the size of the administrative area.

We ran two secondary analyses. First, we repeated the analysis in the restricted set of cities in which we had data on population growth 5 years before the study period, instead of concurrent with the study period, to compare associations of population growth during and before the study period. Second, we ran the analysis of predictors of proportionate mortality in the restricted set of cities with a lower proportion of ill-defined deaths (defined as <90th percentile or 13% ill-defined deaths).

We do not report *P* values to prevent ‘*P* hacking’ and as our goal was not to test hypotheses but rather to report point estimates and levels of precision by the 95% CI.^40^ Data harmonization and cleaning was conducted in R v.4.0.0 and SAS v.9.2. All analyses were conducted in R v.4.0.0 and JAGS v.4.

### Reporting Summary

Further information on research design is available in the Nature Research Reporting Summary linked to this article.

## Online content

Any methods, additional references, Nature Research reporting summaries, source data, extended data, supplementary information, acknowledgements, peer review information; details of author contributions and competing interests; and statements of data and code availability are available at 10.1038/s41591-020-01214-4.

## Supplementary information

Supplementary Information Supplementary Tables 1–8 and Supplementary Figs. 1–3.

Reporting Summary

## Data Availability

Life expectancy and proportionate mortality data with city identifiers are freely available from the interactive app at https://drexel-uhc.shinyapps.io/MS10/. Vital registration data for Brazil, Chile, Colombia and Mexico were downloaded from publicly available repositories from statistical agencies in each country. Vital registration data for Argentina, Costa Rica, El Salvador, Panama and Peru were obtained directly from statistical agencies in each country. A link to these agency websites can be accessed via https://drexel.edu/lac/data-evidence/data-acknowledgements/. Source data are provided with this paper.

## Source data

Source Data Fig. 1 Statistical Source Data. Source Data Fig. 2 Statistical Source Data. Source Data Fig. 3 Statistical Source Data. Source Data Extended Data Fig. 1 Statistical Source Data. Source Data Extended Data Fig. 2 Statistical Source Data. Source Data Extended Data Fig. 3 Statistical Source Data. Source Data Extended Data Fig. 4 Statistical Source Data. Source Data Extended Data Fig. 5 Statistical Source Data. Source Data Extended Data Fig. 6 Statistical Source Data. Source Data Extended Data Fig. 7 Statistical Source Data. Source Data Extended Data Fig. 10 Statistical Source Data.

## Code availability

Code used in this study is available at https://github.com/usamabilal/SALURBAL_MS10.

## References

[1] UNDP Population Division. *World Urbanization Prospects: The 2018 Revision* https://population.un.org/wup/ (2018).

[2] UN-HABITAT. *Urbanization and Development: Emerging Futures; World Cities Report 2016* (UN-HABITAT, 2016).

[3] Fagliano JA, Diez-Roux AV (2018). Climate change, urban health, and the promotion of health equity. PLoS Med..

[4] Whitmee S (2015). Safeguarding human health in the Anthropocene epoch: report of the Rockefeller Foundation–Lancet commission on planetary health. Lancet.

[5] Rydin Y (2012). Shaping cities for health: complexity and the planning of urban environments in the 21st century. Lancet.

[6] Matthews Z (2010). Examining the ‘urban advantage’ in maternal health care in developing countries. PLoS Med..

[7] Dye C (2008). Health and urban living. Science.

[8] Quistberg DA (2019). Building a data platform for cross-country urban health studies: the SALURBAL study. J. Urban Health.

[9] Diez-Roux AV (2018). A novel international partnership for actionable evidence on urban health in Latin America: LAC-urban health and SALURBAL. Glob. Chall..

[10] Mathers CD, Stevens GA, Boerma T, White RA, Tobias MI (2015). Causes of international increases in older age life expectancy. Lancet.

[11] Roth GA (2018). Global, regional, and national age-sex-specific mortality for 282 causes of death in 195 countries and territories, 1980–2017: a systematic analysis for the Global Burden of Disease Study 2017. Lancet.

[12] Naghavi M (2017). Global, regional, and national age-sex specific mortality for 264 causes of death, 1980–2016: a systematic analysis for the Global Burden of Disease Study 2016. Lancet.

[13] World Health Organization. *Global Health Estimates. Disease Burden and Mortality Estimates. Cause-specific Mortality, 2000–2016* https://www.who.int/healthinfo/global_burden_disease/estimates/en/ (2018).

[14] Dandona L (2017). Nations within a nation: variations in epidemiological transition across the states of India, 1990–2016 in the Global Burden of Disease Study. Lancet.

[15] Gómez-Dantés H (2016). Dissonant health transition in the states of Mexico, 1990–2013: a systematic analysis for the Global Burden of Disease Study 2013. Lancet.

[16] Seaman R, Mitchell R, Dundas R, Leyland AH, Popham F (2015). How much of the difference in life expectancy between Scottish cities does deprivation explain?. BMC Public Health.

[17] Andrade F (2009). Estimating diabetes and diabetes-free life expectancy in Mexico and seven major cities in Latin America and the Caribbean. Revista Panamericana de Salud Pública.

[18] United Nations Population Division. *World Population Prospects: The 2019 Revision* https://esa.un.org/unpd/wpp/ (2019).

[19] Franco M, Bilal U, Diez-Roux AV (2015). Preventing non-communicable diseases through structural changes in urban environments. J. Epidemiol. Community Health.

[20] Russell, B. Beyond the local trap: new municipalism and the rise of the fearless cities. *Antipode*10.1111/anti.12520 (2019).

[21] Howe LD (2012). Measuring socio-economic position for epidemiological studies in low- and middle-income countries: a methods of measurement in epidemiology paper. Int. J. Epidemiol..

[22] Konteh FH (2009). Urban sanitation and health in the developing world: reminiscing the nineteenth century industrial nations. Health Place.

[23] Lönnroth K, Jaramillo E, Williams BG, Dye C, Raviglione M (2009). Drivers of tuberculosis epidemics: the role of risk factors and social determinants. Social Sci. Med..

[24] Hastings KG (2018). Socioeconomic differences in the epidemiologic transition from heart disease to cancer as the leading cause of death in the United States, 2003 to 2015: an observational study. Ann. Int. Med..

[25] Rosero-Bixby L (2018). High life expectancy and reversed socioeconomic gradients of elderly people in Mexico and Costa Rica. Demogr. Res..

[26] Omran AR (2005). The epidemiologic transition: a theory of the epidemiology of population change. 1971. Milbank Q.

[27] Miranda JJ (2019). Understanding the rise of cardiometabolic diseases in low- and middle-income countries. Nat. Med..

[28] McKeown RE (2009). The epidemiologic transition: changing patterns of mortality and population dynamics. Am. J. Lifestyle Med..

[29] Bilal U (2019). Inequalities in life expectancy in six large Latin American cities from the SALURBAL study: an ecological analysis. Lancet Planet. Health.

[30] Bettencourt LMA, Lobo J, Helbing D, Kühnert C, West GB (2007). Growth, innovation, scaling, and the pace of life in cities. Proc. Natl Acad. Sci. USA.

[31] Gomez-Lievano A, Youn H, Bettencourt LMA (2012). The statistics of urban scaling and their connection to Zipf’s Law. PloS ONE.

[32] Patz JA (2004). Unhealthy landscapes: policy recommendations on land use change and infectious disease emergence. Env. Health Persp..

[33] Tracey JA, Bevins SN, VandeWoude S, Crooks KR (2014). An agent-based movement model to assess the impact of landscape fragmentation on disease transmission. Ecosphere.

[34] Browning CR (2010). Commercial density, residential concentration, and crime: land use patterns and violence in neighborhood context. J. Res. Crime Delinq..

[35] World Bank. *World Bank Country and Lending Groups* https://datahelpdesk.worldbank.org/knowledgebase/articles/906519 (2017).

[36] Hill K, You D, Choi Y (2009). Death distribution methods for estimating adult mortality: sensitivity analysis with simulated data errors. Demogr. Res..

[37] Palloni, A. & Pinto-Aguirre, G. in *International Handbook of Adult Mortality* (eds Richard G. Rogers & Eileen M. Crimmins) 101–132 (Springer Netherlands, 2011).

[38] Naghavi M (2010). Algorithms for enhancing public health utility of national causes-of-death data. Popul. Health Metr..

[39] Híjar M (2012). Quantifying the underestimated burden of road traffic mortality in Mexico: a comparison of three approaches. Traffic Inj. Prev..

[40] Morgenstern H (1995). Ecologic studies in epidemiology: concepts, principles, and methods. Annu. Rev. Public Health.

[41] Hill, K. Analytical methods to evaluate the completeness and quality of death registration: Current state of knowledge. *Population Division Technical Paper**2017/2* (United Nations, 2017).

[42] Adair T, Lopez AD (2018). Estimating the completeness of death registration: an empirical method. PloS ONE.

[43] Murray CJ, Rajaratnam JK, Marcus J, Laakso T, Lopez AD (2010). What can we conclude from death registration? Improved methods for evaluating completeness. PLoS Med..

[44] Hill, K. Estimating census and death registration completeness. *Asian Pac. Popul. Forum.* **1**, 8–13, 23–24 (1987). 12280697

[45] Bennett NG, Horiuchi S (1984). Mortality estimation from registered deaths in less developed countries. Demography.

[46] Peralta A (2019). Evaluation of the mortality registry in Ecuador (2001–2013) – social and geographical inequalities in completeness and quality. Popul. Health Metr..

[47] DDM: Death Registration Coverage Estimation (R Package) (2017).

[48] Schmertmann CP, Gonzaga MR (2018). Bayesian estimation of age-specific mortality and life expectancy for small areas with defective vital records. Demography.

[49] Gonzaga MR, Schmertmann CP (2016). Estimating age-and sex-specific mortality rates for small areas with TOPALS regression: an application to Brazil in 2010. Revista Brasileira de Estudos de População.

[50] Kashnitsky, I. & Riffe, T. DemoTools (R package) (2019).

[51] World Health Organization. *Global Health Estimates: Deaths By Cause, Age, Sex And Country, 2000–2012.* **9** (WHO, 2014).

[52] Office for National Statistics. *Method Changes to Life and Health State Expectancies* (ONS, 2016).

[53] Rubin, D. B. *Multiple Imputation for Nonresponse in Surveys*. Vol. 81 (John Wiley & Sons, 2004).

