# Supplementary material for: Life expectancy and mortality in 363 cities of Latin America
Source: Nat Med. 2021 Jan 25;27(3):463–70. doi: 10.1038/s41591-020-01214-4 (PMC7960508; doi:10.1038/s41591-020-01214-4)
Supplement: Supplementary file 1 — Supplementary Tables 1–8 and Supplementary Figs. 1–3. [file 41591_2020_1214_MOESM1_ESM.pdf]

nature research

https://doi.org/10.1038/s41591-020-01214-4

Supplementary information

Life expectancy and mortality in 363 cities of Latin America

In the format provided by the authors and unedited

Supplementary Information

Life expectancy and mortality in 363 cities of Latin America: the SALURBAL study

Supplementary Table 1: Variance partitioning for life expectancy between iterations, cities and countries at different ages for men and women

	Women			Men		
**Age**	**Iteration**	**City**	**Country**	**Iteration**	**City**	**Country**
**Birth**	4.0%	41.3%	54.7%	3.0%	45.5%	51.5%
**20**	4.0%	39.1%	56.9%	3.0%	40.9%	56.1%
**40**	4.0%	38.1%	57.9%	3.9%	33.4%	62.7%
**60**	4.3%	38.3%	57.3%	4.7%	33.4%	62.0%

Footnote: calculated with a linear mixed model of life expectancy, with a random intercept for country and city.

Supplementary Table 2: Proportionate mortality by country and cause (redistributed), and proportion of ill-defined diseases and injuries of ill-defined intent.

Country	# Cities	CMNN	Cancer	CVD/NCDs	Unintentional Injuries	Violent Injuries	Ill-defined Diseases	Injuries of Ill-defined Intent
**Argentina**	33	15.7% [10;24.1]	20% [15.6;26.5]	57.8% [48.3;62.5]	4.2% [2.7;7.3]	2.2% [1.4;4]	6% [0.4;31.8]	0% [0.03;4.06]
**Brazil**	152	14.2% [6.5;19.6]	18.4% [9.2;25.8]	54.5% [40.7;62.9]	6.2% [3.6;15.1]	6.7% [1.5;20.4]	4.3% [0.3;23]	0% [0.07;27.72]
**Chile**	21	7.9% [6;9.6]	26.7% [24.4;30.5]	58.6% [52.2;61.7]	4.4% [3.5;7.8]	2.5% [1.7;4.6]	2.1% [1.4;4.6]	0% [0;0]
**Colombia**	35	10.1% [6.3;23.2]	21.8% [12.9;26]	55.6% [44.7;65.9]	5.1% [3.4;14.2]	7.4% [3.2;16.1]	1.4% [0.2;4.4]	0% [0.6;2.9]
**Costa Rica**	1	7%	25.30%	58%	5.80%	3.90%	1.50%	0%
**Mexico**	92	9.8% [6.9;17]	13.7% [9.7;17.8]	65.1% [52.4;71.3]	6% [3.9;12.3]	5.4% [1.7;19]	0.8% [0.2;5.4]	0% [0.08;6.54]
**Panama**	3	13.6% [11.7;17.5]	18.6% [16;19.1]	57.3% [54.8;61.8]	5.1% [4.9;6]	5.5% [2.5;6.2]	1.8% [1.1;2.1]	0% [1.06;1.54]
**Peru**	23	25.7% [21.1;54.9]	21.3% [9;25.6]	44.5% [27.7;54.6]	7.3% [3.8;19.2]	1.2% [0.1;2.8]	0.6% [0.1;3.5]	0% [0.09;3.32]
**El Salvador**	3	14.6% [13.1;14.9]	15.8% [13.1;16.5]	54.8% [53.5;61.3]	4.8% [4.5;5.2]	10% [8.2;10.2]	15% [11.9;27.5]	0% [0.24;0.52]
**Overall**	363	13.1% [6;54.9]	18.2% [9;30.5]	57.5% [27.7;71.3]	5.7% [2.7;19.2]	5.4% [0.1;20.4]	3.2% [0.1;31.8]	0% [0;27.72]

Footnote: Proportions represent proportionate mortality for all cities of a given country. In brackets are [minimum;maximum] proportions by city for each country. Costa Rica only has one city, so all values refer to the % PM (or ill-defined deaths) in the only city. CMNN: communicable, maternal, neonatal and nutritional conditions; CVD/NCDs: cardiovascular disease and other non-communicable diseases.

Supplementary Table 3: Variability in proportionate mortality within and between countries

	Raw		Age-adjusted	
**Cause of Death**	**% Variability Between Countries**	**% Variability Within Countries**	**% Variability Between Countries**	**% Variability Within Countries**
**CMNN**	80%	20%	82%	18%
**Cancer**	71%	29%	74%	26%
**CVD/NCDs**	64%	36%	74%	26%
**Unintentional**	38%	62%	24%	76%
**Violent**	38%	62%	48%	52%

Footnote: % Variability between countries is the intraclass correlation coefficient of a multilevel linear regression of proportionate mortality by each cause with no predictor, and a random intercept for country. % Variability within countries is the complementary (1-ICC). The age-adjusted model is conducted using age-adjusted proportionate mortality (% of all-cause age-adjusted mortality due to each cause-specific age-adjusted mortality).

Supplementary Table 4: Indicators, definitions and sources of city characteristics used in this study

Indicator	Definition	Interpretation (Higher value means…)	Source
Age-Adjusted Mortality	Deaths per 100,000 people, adjusted using the WHO 2000 world population	Cities with higher levels of mortality	Vital Statistics and Population Projections
City Size	Population size within the administrative boundaries of the city at baseline	Cities with more population	Populations Projections
Population Growth	Change in population size during the study period	Cities that have grown more in population	Populations Projections
Population Density	Population per square kilometers in the administrative area in 2010	Denser urban development	Worldpop
Urban Extent Fragmentation	Number of urban patches in the administrative area divided by the total area (in km2)	Higher fragmentation of urban expansion	Global Urban Footprint project
Street Connectivity	Number of intersections per km2 in the administrative area	More connectivity between streets	OpenStreetMap
Education Levels	Proportion of the population aged 25 or above with completed primary education or above	Higher average educational achievement	Census
Water Access	Proportion of households with access to piped water	Higher access to water	Census
Sanitation	Proportion of households with connection with the municipal sewage network	Higher levels of sanitation	Census
Overcrowding	Proportion of households with more than 3 people per room	Higher overcrowding	Census
Social Environment Index	Composite index of the four social indicators (with overcrowding reversed)	Higher socioeconomic development	Census (composite)

Footnote: variables were calculated by the SALURBAL team using data from the referred source. Appendix Table 1.2 contains detailed data on the year for each variable-country. For more details, please check the article by Quistberg et al (see references of manuscript).

Supplementary Table 5: Years of Data for every indicator-country

Indicator	Source	AR	BR	CL	CO	CR	MX	PA	PE	SV
Life Expectancy	Vital Statistics and Population Projections	2012-2016	2012-2016	2012-2016	2012-2016	2012-2016	2012-2016	2012-2016	2012-2016	2010-2014
Mortality by cause	Vital Statistics	2012-2016	2012-2016	2012-2016	2012-2016	2012-2016	2012-2016	2012-2016	2012-2016	2010-2014
Age-Adjusted Mortality	Vital Statistics and Population Projections	2012-2016	2012-2016	2012-2016	2012-2016	2012-2016	2012-2016	2012-2016	2012-2016	2010-2014
City Size	Populations Projections	2012	2012	2012	2012	2012	2012	2012	2012	2010
City Growth	Populations Projections	2012-2016	2012-2016	2012-2016	2012-2016	2012-2016	2012-2016	2012-2016	2012-2016	2010-2014
Population Density	Worldpop	2010	2010	2010	2010	2010	2010	2010	2010	2010
Urban Patch Density	Global Urban Footprint project	2012	2012	2012	2012	2012	2012	2012	2012	2012
Intersection Density	OpenStreetMap	2017	2017	2017	2017	2017	2017	2017	2017	2017
Education Levels	Census	2010	2010	2002	2005	2011	2010	2010	2007	2007
Water Access	Census	2010	2010	2002	2005	2011	2010	2010	2007	2007
Sewage Connection	Census	2010	2010	2002	2005	2011	2010	2010	2007	2007
Overcrowding	Census	2010	2010	2002	2005	2011	2010	2010	2007	2007

Footnote: AR: Argentina, BR: Brazil, CL: Chile, CO: Colombia, CR: Costa Rica, MX: Mexico, PA: Panama, PE:Peru, SV: El Salvador

Supplementary Table 6: detailed description of population denominators.

Country	Source	Latest Census	Type[a]	1-4 age group[b]	Open Age Group[c]
Argentina	INDEC	2010	Projections	No	80+
Brazil	IBGE	2010	Projections	No	80+
Chile	INE	2017	Estimations	Yes	80+
Colombia	DANE	2018	Estimations	Yes	85+
Costa Rica	INEC	2011	Projections	No	75+
Mexico[d]	CONAPO/SALURBAL	2010/15	Mixed	Mixed	Mixed
Panama	INEC	2010	Projections	No	80+
Peru[e]	INEI	2017	Projections	No	80+
El Salvador[f]	DIGESTYC/SALURBAL	2007	Projections	No	80+

Footnote: details on sources are available on the SALURBAL study protocol by Quistberg et al (see references of manuscript).

a) Projections refer to population projections created by the country statistical offices/census bureaus based on the latest available census. Estimations refer to intercensal estimations created by the contry statistical offices/census bureaus or the SALURBAL team.
b) When 1-4 age group data was not available, we disaggregated the 0-4 group into 0-1 and 1-4 using a penalized composite link model.
c) Open age groups below 85+ were disaggregated up to 85+ using a penalized composite link model. We used an upper age limit of 120 years and created a new open-ended age group at 85+.
d) Mexico: We conducted a linear interpolation between the population projections created by CONAPO based on the 2010 census and the 2015 intercensal survey, by age group, sex and municipality. We also used 2016 data from the 2015 version of the population projections. The 2010 projections had the 1-4 age group disaggregated and an open ended age group of 85+, while the 2015 ones did not disaggregate the 1-4 age group and had an open ended age group of 65+. We disaggregated the 1-4 age group and disaggregated the open-ended group to 85+ using a penalized composite link model.
e) Peru: at the time of this article, Peru had no population estimations based on the 2017 census. We used population projections based on the 2007 census.
f) El Salvador: We conducted a linear interpolation between the 2007 census and the 2015 population projections created by DIGESTYC, by age group, sex and municipality. The 2010 projections had the 1-4 age group disaggregated and an open ended age group of 85+, while the 2015 ones did not disaggregate the 1-4 age group and had an open ended age group of 65+. We disaggregated the 1-4 age group and disaggregated the open-ended group to 85+ using a penalized composite link model.

Supplementary Table 7: Descriptive statistics of city-Level factors of 363 Latin American Cities by size

City Size	Overall	100-250k	>=250-500k	>=500-1M	>=1M-5M	>=5M
# Cities	363	161	96	59	40	7
Life Expectancy at birth (men)	71.9 [70.2;73.4]	71.8 [70.4;73.4]	71.9 [70.1;73.3]	72.4 [70.3;73.5]	71.4 [69.4;72.8]	73.0 [72.4;76.5]
Life Expectancy at birth (women)	78.4 [77.3;79.6]	78.4 [77.2;79.6]	78.2 [77.2;79.1]	78.5 [77.3;79.9]	78.6 [77.8;79.8]	79.5 [78.7;80.8]
% CMNN+	12.6 [9.8;15.5]	13.0 [10.4;15.7]	12.7 [9.5;16.6]	11.8 [9.5;14.9]	12.5 [10.1;14.2]	14.2 [8.6;16.2]
% Cancer	17.8 [14.4;20.4]	18.0 [14.1;20.5]	17.4 [14.5;20.6]	17.5 [14.4;20.6]	17.3 [14.6;19.8]	19.4 [18.2;23.8]
% CVD/NCDs^	56.2 [53.0;60.4]	56.3 [52.8;60.0]	56.0 [52.7;60.1]	56.3 [53.3;63.0]	55.7 [53.1;62.4]	57.0 [56.4;59.3]
% Unintentional Injuries	6.4 [5.4;7.5]	6.7 [5.5;7.6]	6.2 [5.4;7.5]	6.5 [5.4;7.6]	5.6 [4.9;6.6]	5.0 [4.3;5.5]
% Violent Injuries	4.5 [2.9;7.6]	4.2 [2.9;7.5]	4.6 [2.6;7.5]	4.4 [3.3;6.5]	7.3 [3.9;10.3]	4.0 [2.3;4.6]
% Ill-defined diseases	2.0 [0.9;4.7]	2.6 [1.4;5.8]	1.9 [0.8;4.9]	1.4 [0.8;2.7]	1.7 [0.9;3.0]	1.9 [1.2;2.8]
% Injuries of Ill-defined intent	0.9 [0.4;1.6]	0.7 [0.4;1.4]	1.0 [0.4;1.6]	1.2 [0.5;1.7]	1.1 [0.6;1.7]	2.0 [1.5;2.1]
AAMR* (deaths/100,000)	623.1 [563.0;690.0]	625.2 [567.7;690.5]	626.7 [579.1;694.4]	602.8 [543.9;689.6]	632.2 [552.1;675.1]	582.8 [461.5;596.3]
City Size (million)	0.3 [0.2;0.6]	0.2 [0.1;0.2]	0.3 [0.3;0.4]	0.7 [0.6;0.9]	1.8 [1.3;3.0]	12.1 [9.0;17.7]
City Growth (%/5 years)	4.7 [3.5;6.4]	4.5 [3.4;6.2]	4.8 [3.6;6.7]	5.1 [4.0;6.4]	4.6 [3.2;6.2]	3.5 [3.2;4.2]
Pop. Density (1000 pop/km2)	6.1 [5.0;8.3]	5.5 [4.6;7.9]	6.6 [5.2;8.7]	6.0 [5.1;8.2]	6.9 [5.6;8.4]	10.7 [9.5;13.3]
Fragmentation (patches/km2)	0.3 [0.1;0.6]	0.23 [0.08;0.41]	0.2 [0.1;0.4]	0.4 [0.2;0.6]	0.6 [0.4;0.8]	0.6 [0.5;0.8]
Street Connectivity (int./km2)	4.6 [2.0;8.9]	3.4 [1.5;6.2]	4.1 [2.2;7.2]	7.0 [3.2;11.3]	14.0 [8.8;18.1]	28.0 [22.5;37.2]
Primary Education (%)	72.9 [66.0;80.0]	69.0 [63.5;77.4]	74.4 [66.7;79.0]	77.8 [71.1;82.3]	76.9 [71.2;82.0]	85.6 [77.3;86.1]
Piped water in household (%)	92.1 [81.1;97.3]	93.8 [82.7;97.9]	89.1 [73.7;96.0]	91.1 [79.2;96.9]	93.8 [87.2;96.0]	95.3 [86.0;96.7]
Sewage network connection (%)	77.3 [55.4;89.9]	74.1 [52.1;89.2]	76.4 [58.2;90.0]	82.1 [63.3;90.3]	78.3 [45.6;87.8]	88.0 [82.7;93.8]
Overcrowded household (%)	4.2 [2.5;8.7]	3.4 [1.9;7.4]	5.5 [2.9;10.0]	5.6 [2.9;8.9]	3.6 [2.9;6.7]	3.9 [3.5;7.8]
Social Environment Index	0.1 [-0.3; 0.5]	0.08 [-0.31; 0.49]	0.04 [-0.51; 0.47]	0.21 [-0.03; 0.55]	0.4 [-0.1; 0.5]	0.3 [0.3;0.7]

Footnote: All measures are medians [IQR]. Social Environment Index is a composite index calculated from z-scores of primary education, piped water, and overcrowding (reversed). *:Age-adjusted mortality rate.^: Cardiovascular Diseases and other Non-Communicable Diseases. +:Communicable, Maternal, Neonatal and Nutritional.

Supplementary Table 8: ICD10 codes included for each category

Category	ICD10 codes
CMNN	A00-B99, G00-G04, G14, N70-N73, J00-J06, J09-J18, J20-J22, H65-H66, O00-O99, P00-P96, E00-E02, E40-E46, E50, D50-D53, D64.9, E51-E64
Cancer	C00-C97, D00-D48
CVD/NCDs	D55-D64 (except D64.9) D65-D89, E03-E07, E10-E16, E20-E34, E65-E88, F01-F99, G06-G98 (except G14), H00-H61, H68-H93, I00-I99, J30-J98, K00-K92, N00-N64, N75-N98, L00-L98, M00-M99, Q00-Q99, X41, X42, X45, R95
Unintentional injuries	V01-X59 (except X41, X42, X45), Y40-Y86, Y88, Y89
Violent injuries	X60-Y09, Y35-Y36, Y870, Y871
Ill-defined diseases	R00-R94, R96-R99
Injuries of ill-defined intent	Y10-Y34, Y872

Supplementary Figure 1: Relative Standard Errors of life expectancy (LE).

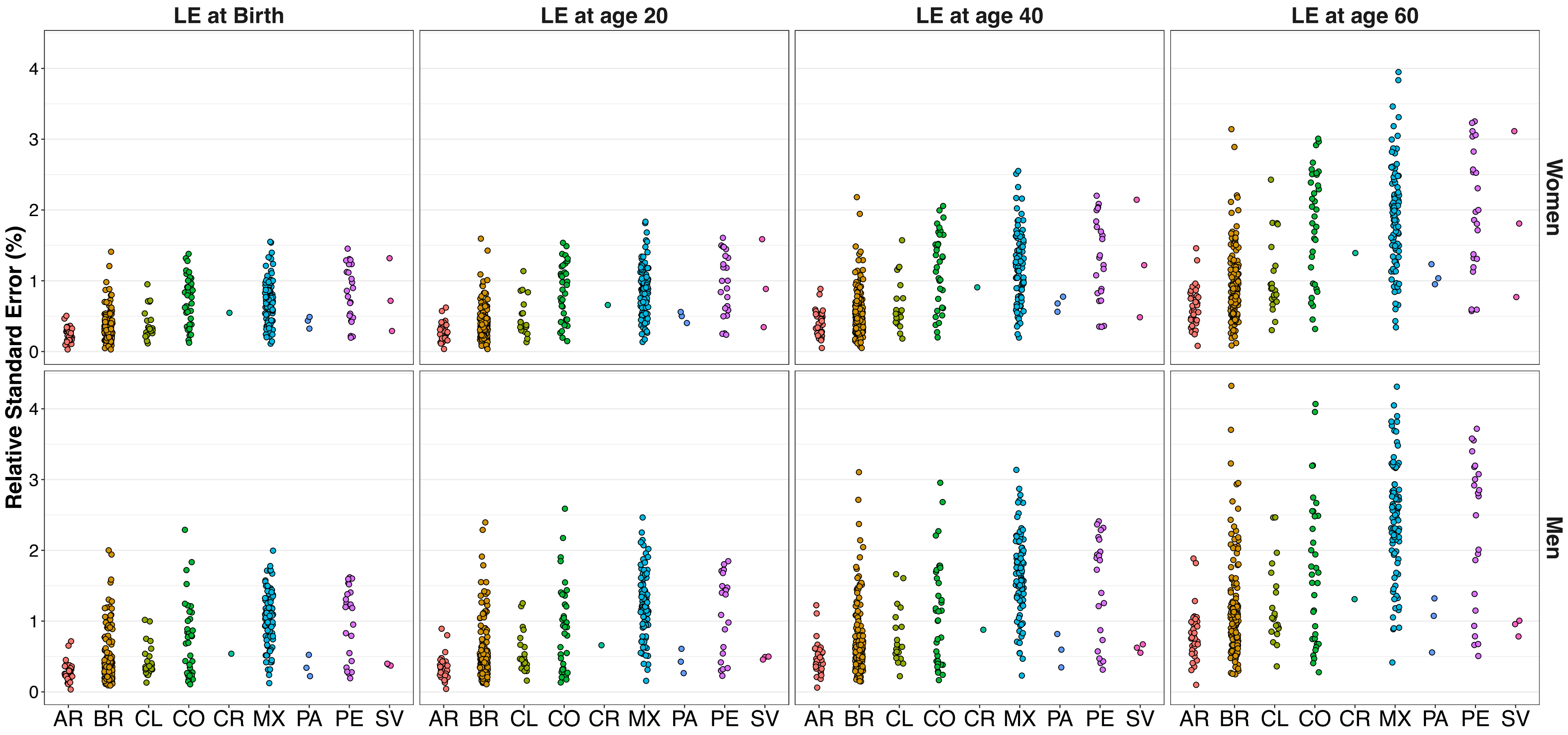

Footnote: Relative standard error=SE/median * 100. AR (Argentina), BR (Brazil), CL (Chile), CO (Colombia), CR (Costa Rica), MX (Mexico), PA (Panama), PE (Peru), SV (El Salvador). LE=life expectancy.

Supplementary Figure 2: estimates of completeness at the city level

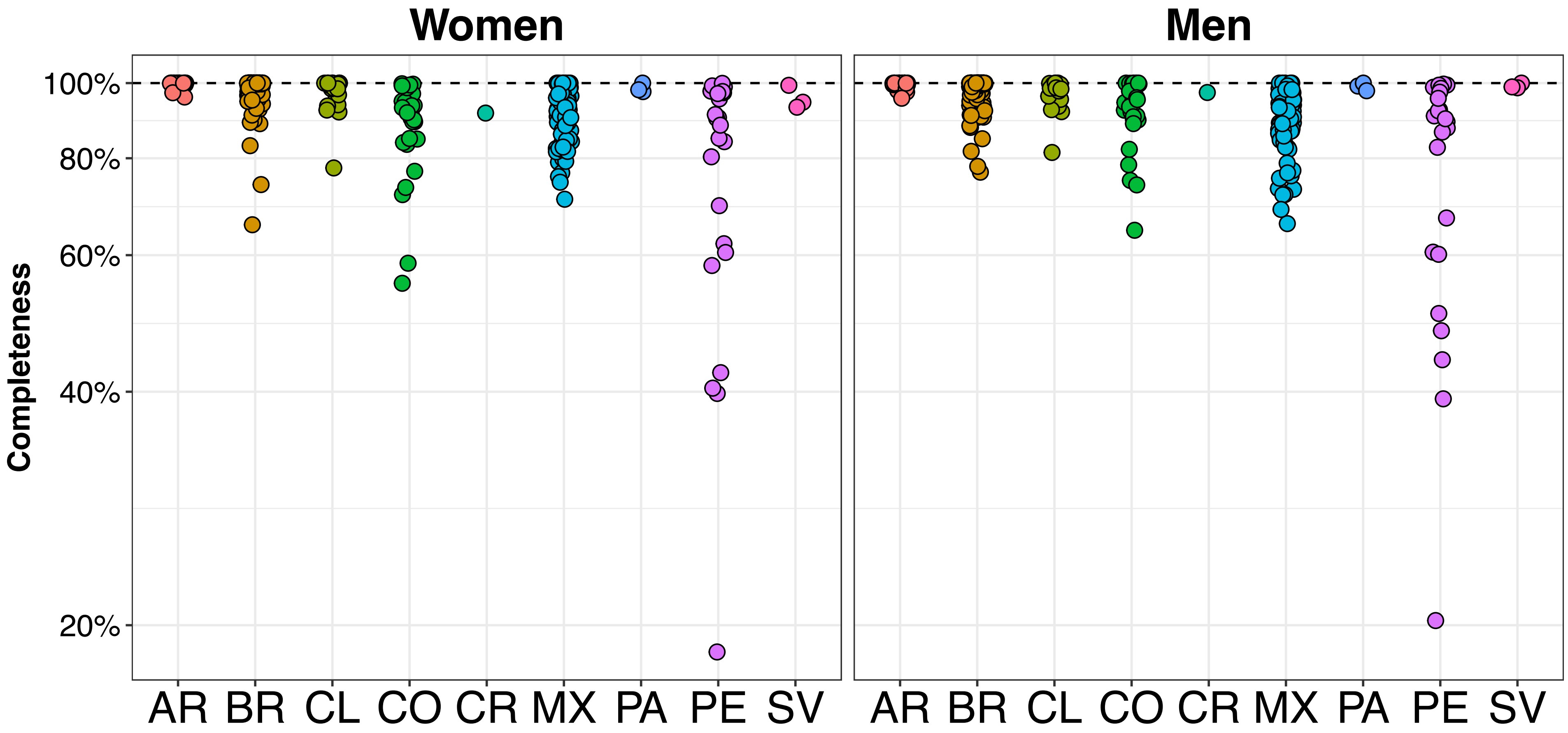

Footnote: Completeness is defined as the % of deaths that are registered in a given city. See methods for a more detailed explanation of the methods used to estimate undercounting. AR (Argentina), BR (Brazil), CL (Chile), CO (Colombia), CR (Costa Rica), MX (Mexico), PA (Panama), PE (Peru), SV (El Salvador)

Supplementary Figure 3: Proportion deaths due to ill-defined deaths and injuries of ill-defined intent, by country.

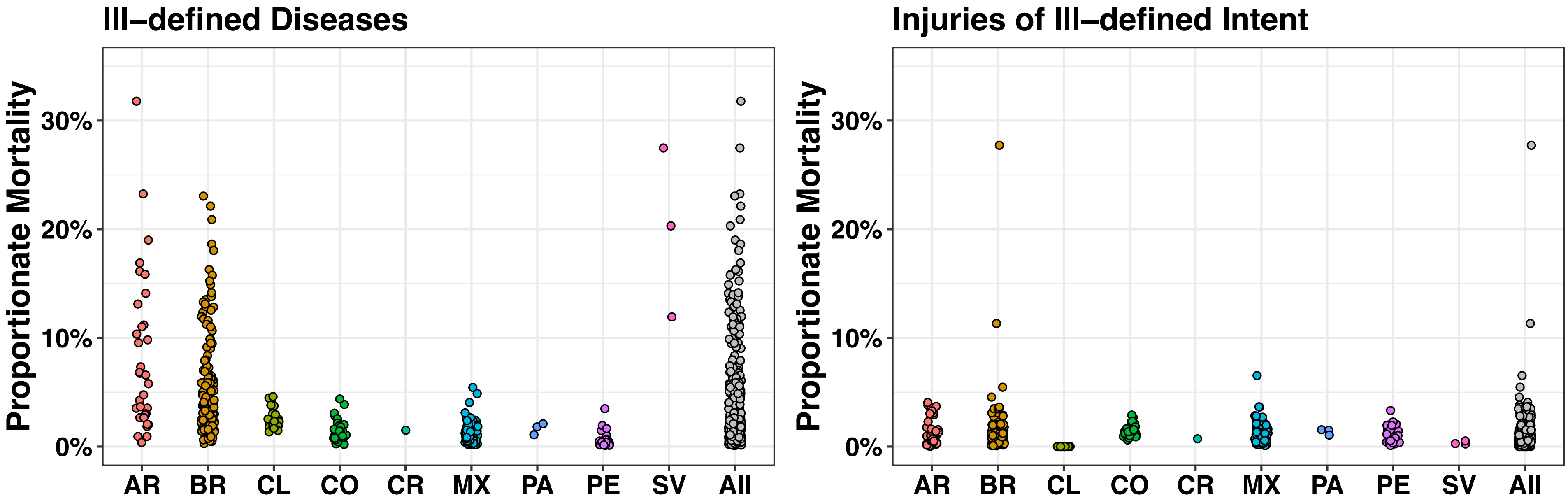

Footnote: AR (Argentina), BR (Brazil), CL (Chile), CO (Colombia), CR (Costa Rica), MX (Mexico), PA (Panama), PE (Peru), SV (El Salvador)
