# Supplementary material for: Life expectancy and mortality in 363 cities of Latin America
Source: Nat Med. 2021 Jan 25;27(3):463–70. doi: 10.1038/s41591-020-01214-4 (PMC7960508; doi:10.1038/s41591-020-01214-4)
Supplement: Supplementary file 2 — Reporting Summary [file 41591_2020_1214_MOESM2_ESM.pdf]

nature research

Corresponding author(s): Usama Bilal

Last updated by author(s): Dec 10, 2020

Reporting Summary

Nature Research wishes to improve the reproducibility of the work that we publish. This form provides structure for consistency and transparency in reporting. For further information on Nature Research policies, see Authors & Referees and the Editorial Policy Checklist.

Statistics

For all statistical analyses, confirm that the following items are present in the figure legend, table legend, main text, or Methods section.

n/a	Confirmed	
☐	☒	The exact sample size (*n*) for each experimental group/condition, given as a discrete number and unit of measurement
☐	☒	A statement on whether measurements were taken from distinct samples or whether the same sample was measured repeatedly
☒	☐	The statistical test(s) used AND whether they are one- or two-sided<br>*Only common tests should be described solely by name; describe more complex techniques in the Methods section.*
☐	☒	A description of all covariates tested
☐	☒	A description of any assumptions or corrections, such as tests of normality and adjustment for multiple comparisons
☐	☒	A full description of the statistical parameters including central tendency (e.g. means) or other basic estimates (e.g. regression coefficient) AND variation (e.g. standard deviation) or associated estimates of uncertainty (e.g. confidence intervals)
☒	☐	For null hypothesis testing, the test statistic (e.g. *F*, *t*, *r*) with confidence intervals, effect sizes, degrees of freedom and *P* value noted<br>*Give P values as exact values whenever suitable.*
☐	☒	For Bayesian analysis, information on the choice of priors and Markov chain Monte Carlo settings
☐	☒	For hierarchical and complex designs, identification of the appropriate level for tests and full reporting of outcomes
☐	☒	Estimates of effect sizes (e.g. Cohen's *d*, Pearson's *r*), indicating how they were calculated

Our web collection on statistics for biologists contains articles on many of the points above.

Software and code

Policy information about availability of computer code

Data collection	R v4.0.0 and SAS v9.2 was used to harmonize data provided by the statistical agencies.
Data analysis	R v4.0.0, JAGS v4, R Packages DDM (v1.0.0) & DemoTools (v01.06.01). Code available: https://github.com/usamabilal/SALURBAL_MS10

For manuscripts utilizing custom algorithms or software that are central to the research but not yet described in published literature, software must be made available to editors/reviewers. We strongly encourage code deposition in a community repository (e.g. GitHub). See the Nature Research guidelines for submitting code & software for further information.

Data

Policy information about availability of data

All manuscripts must include a data availability statement. This statement should provide the following information, where applicable:

- Accession codes, unique identifiers, or web links for publicly available datasets
- A list of figures that have associated raw data
- A description of any restrictions on data availability

Life expectancy and proportionate mortality data with city identifiers are freely available from the interactive app at https://drexel-uhc.shinyapps.io/MS10/. SALURBAL acknowledges the contributions of many different agencies in generating, processing, facilitating access to data or assisting with other aspects of the project. Vital registration data for Brazil, Chile, Colombia, and Mexico was downloaded from publicly available repositories from statistical agencies in each country (IBGE, INE, DANE, INEGI), while vital registration data for Argentina, Costa Rica, El Salvador, Panama and Peru was obtained directly from statistical agencies in each country (INDEC, INEC, DGIS, INEC, INEI). For a link to these agencies website, see here: https://drexel.edu/lac/data-evidence/data-acknowledgements/. The SALURBAL project welcomes queries from anyone interested in learning more about its dataset and accessing its data. To learn more about SALURBAL's datasets, visit https://drexel.edu/lac/ or contact the project at salurbal@drexel.edu.

Field-specific reporting

Please select the one below that is the best fit for your research. If you are not sure, read the appropriate sections before making your selection.

☐ Life sciences ☒ Behavioural & social sciences ☐ Ecological, evolutionary & environmental sciences

For a reference copy of the document with all sections, see nature.com/documents/nr-reporting-summary-flat.pdf

Behavioural & social sciences study design

All studies must disclose on these points even when the disclosure is negative.

Study description	Quantitative cross-sectional study examining heterogeneity in life expectancy and proportionate mortality across Latin American cities, and the predictors of both life expectancy and proportionate mortality.
Research sample	The universe of cities above 100,000 people in 9 Latin American countries. Every urban agglomeration with more than 100,000 people was included (as a commonly used threshold to define a city). We included everyone in these cities, as these analysis use universal vital registration data (no restrictions based on age, sex, etc.)
Sampling strategy	No sampling was used, as the entire universe of cities under the inclusion criteria was used. Moreover, the vital registration data is universal by nature. When issues with undercounting in vital registration were present, appropriate demographic methods were employed (see Methods section)
Data collection	Data was compiled by national statistical agencies as part of their vital registration systems. We were responsible of harmonizing this data to make it comparable across the countries in the study.
Timing	Data from years 2010 to 2016 was used. Specifically, we used data for 2010-2014 for El Salvador and 2012-2016 or all other countries. All deaths occurring in these days were included.
Data exclusions	We used all data available for all the 363 cities in the 9 countries.
Non-participation	All cities were included.
Randomization	No randomization, observational study. The first model includes each covariate individually with no adjustment. The multivariable model includes all covariables listed in the corresponding table.

Reporting for specific materials, systems and methods

We require information from authors about some types of materials, experimental systems and methods used in many studies. Here, indicate whether each material, system or method listed is relevant to your study. If you are not sure if a list item applies to your research, read the appropriate section before selecting a response.

Materials & experimental systems

n/a	Involved in the study
☒	☐ Antibodies
☒	☐ Eukaryotic cell lines
☒	☐ Palaeontology
☒	☐ Animals and other organisms
☐	☒ Human research participants
☒	☐ Clinical data

Methods

n/a	Involved in the study
☒	☐ ChIP-seq
☒	☐ Flow cytometry
☒	☐ MRI-based neuroimaging

Human research participants

Policy information about studies involving human research participants

Population characteristics	We included the entire population covered by the vital registration system of each city. We have made no restrictions based on age, sex, or any other characteristics. Due to completeness issues, vital registration may not cover the entire population. We have applied demographic death distribution methods to correct this issue (see methods for details).
Recruitment	We included the entire universe covered by vital registration. It is possible that some deaths may not be included due to completeness issues. To address this have applied demographic death distribution methods to correct this issue (see methods for details).
Ethics oversight	The SALURBAL study protocol was approved by the Drexel University Institutional Review Board with ID #1612005035.

Note that full information on the approval of the study protocol must also be provided in the manuscript.
